# Sex-specific genetic effects associated with pigmentation, sensitivity to sunlight, and melanoma in a population of Spanish origin

**DOI:** 10.1186/s13293-016-0070-1

**Published:** 2016-03-18

**Authors:** Barbara Hernando, Maider Ibarrola-Villava, Lara P. Fernandez, Maria Peña-Chilet, Marta Llorca-Cardeñosa, Sara S. Oltra, Santos Alonso, Maria Dolores Boyano, Conrado Martinez-Cadenas, Gloria Ribas

**Affiliations:** Department of Medicine, Jaume I University of Castellon, Av. Sos Baynat s/n, 12071 Castellon, Spain; Department of Medical Oncology, Biomedical Research Institute - INCLIVA, University of Valencia, Av. Menendez Pelayo 4 accesorio, 46010 Valencia, Spain; Molecular Oncology and Nutritional Genomics of Cancer Group, IMDEA Food Institute, CEI UAM + CSIC, Madrid, Spain; Department of Genetics, Physical Anthropology and Animal Physiology, University of the Basque Country UPV/EHU, Leioa, Bizkaia Spain; Department of Cell Biology and Histology, University of the Basque Country UPV/EHU, Leioa, Bizkaia Spain; BioCruces Health Research Institute, Cruces University Hospital, Cruces-Barakaldo, Bizkaia Spain

**Keywords:** Pigmentation, UV sensitivity, Skin cancer, Sex, Polymorphisms

## Abstract

**Background:**

Human pigmentation is a polygenic quantitative trait with high heritability. In addition to genetic factors, it has been shown that pigmentation can be modulated by oestrogens and androgens via up- or down-regulation of melanin synthesis. Our aim was to identify possible sex differences in pigmentation phenotype as well as in melanoma association in a melanoma case-control population of Spanish origin.

**Methods:**

Five hundred and ninety-nine females (316 melanoma cases and 283 controls) and 458 males (234 melanoma cases and 224 controls) were analysed. We genotyped 363 polymorphisms (single nucleotide polymorphisms (SNPs)) from 65 pigmentation gene regions.

**Results:**

When samples were stratified by sex, we observed more SNPs associated with dark pigmentation and good sun tolerance in females than in males (107 versus 75; *P* = 2.32 × 10^−6^), who were instead associated with light pigmentation and poor sun tolerance. Furthermore, six SNPs in *TYR*, *SILV/CDK2*, *GPR143*, and *F2RL1* showed strong differences in melanoma risk by sex (*P* < 0.01).

**Conclusions:**

We demonstrate that these genetic variants are important for pigmentation as well as for melanoma risk, and also provide suggestive evidence for potential differences in genetic effects by sex.

**Electronic supplementary material:**

The online version of this article (doi:10.1186/s13293-016-0070-1) contains supplementary material, which is available to authorized users.

## Background

Human pigmentation traits are some of the most visible and differentiable human characteristics. Pigmentation in human tissue is attributable to the number, size and cellular distribution of melanosomes produced, and the type of melanin synthesised (the black-brown coloured eumelanin or the red-yellow coloured phaeomelanin), while the number of melanocytes is usually unchanged [1].

The type of melanin synthesised is influenced by sun exposure and is genetically controlled [2]. Ultraviolet (UV) exposure plays a key role in the evolutionary selective pressure on human pigmentation. Geographic distribution of human skin pigmentation reflects an adaptation to latitude-dependent levels of UV radiation [3, 4]. The linear relationship between worldwide skin pigmentation variation, latitude, and UV radiation levels is thought to result from the physiological role of melanin type in UV-mediated vitamin D synthesis, UV-induced photolysis of folate, and in the protection from exposure to UV, which can cause sunburn and skin cancer [5]. However, the physiological role for eye and hair colour variations still remains unknown.

Variation in genes implicated in human pigmentation has been associated with phenotypic characteristics such as skin colour, hair colour, eye colour, freckling, and sensitivity to sunlight [6], and also with the risk of various types of skin cancer [7–15]. The proteins encoded by these genes have effects at various stages of the pigmentation pathway, ranging from melanogenesis, the stabilisation and transport of enzymes in the melanin production pathway, the production and maintenance of melanosomes and the melanosomal environment, and the switch between the production of eumelanin and phaeomelanin. Other pigmentation-related proteins code for intrinsic factors that help in the regulation of pigmentation, such as factors produced by fibroblasts in the dermis that affect overlying melanocytes and keratinocytes, and endocrine factors from the blood supply, as well as neural factors and inflammation-related factors [6, 16, 17].

Melanin synthesis is also modulated, in part, by oestrogens and androgens [18]. Physiological hyperpigmentation in various forms (tanning, dark spots, chloasma, linea nigra, and/or melasma) is commonly seen in pregnant females due to an increase of the levels of pregnancy-related hormones [18]. The increase of pregnancy-related hormones—oestrogen, progesterone, and melanocyte-stimulating hormone (α-MSH)—during gestation induces the activation and expression of genes involved in melanin synthesis in melanocytes [19], while it has also been shown that androgens inhibit tyrosinase activity [20]. In addition to sex-endocrine factors, the use of oestrogen-containing oral contraceptives, certain cosmetics, and oestrogen-progesterone therapies has also been associated with hyperpigmentation [21].

Biological and behavioural gender differences likely contribute to the sexual disparity in skin aging, pigmentation, and melanoma incidence and outcome [22, 23]. Recent studies point to Caucasian females having slightly darker eye colour [24, 25] and skin colour [26] than Caucasian males. Regarding melanoma, females show lower melanoma predisposition and incidence, lower risk of metastases, and longer melanoma-specific survival rates than males [27, 28]. Anatomic location of melanoma indeed tends to be different between sexes, being most commonly on the lower leg, hip, and thigh in females and on the back, abdomen, and chest in males [27].

In order to reveal possible sex-related differences in pigmentation phenotype as well as in melanoma association, we investigated the influence of 363 polymorphisms from 65 gene regions—previously associated with pigmentation traits, congenital pigmentation genetic syndromes, and/or skin cancer—in a melanoma case-control population of Spanish origin.

## Methods

### Study subjects and data collection

In this study, a total number of 599 females (316 melanoma cases and 283 cancer-free controls) and 458 males (234 melanoma cases and 224 cancer-free controls) were collected at several Spanish hospitals. We carefully selected all cases and controls included in the current study to account for confounding variables. All individuals were Caucasians of Spanish origin where, according to a previous work by Laayouni and cols, there is no evidence of genetic heterogeneity within different Spanish geographical regions [29]. Controls were frequency-matched to the cases by age and place of birth.

A standardised questionnaire was used to collect information on sex, age, pigmentation characteristics (eye colour, hair colour, skin colour, number of naevi, and presence of solar lentigines), history of childhood sunburns, Fitzpatrick’s skin type classification, and personal and family history of cancer, to classify individuals as previously described [30]. Forty melanoma cases from our previous work were excluded in the current analysis due to the absence of sex details.

All individuals gave written informed consent and the study was approved by the Ethics Committee of the Gregorio Marañon Hospital (Madrid, Spain) and the Biomedical Research Institute - INCLIVA (Valencia, Spain).

### Gene, SNP selection, and genotyping

Gene and single nucleotide polymorphism (SNP) selection was performed as previously described [30]. Sixty-five gene regions were included in this study. They covered a broad range of biological processes, mostly related to pigmentation. We genotyped a total number of 384 tag-SNPs from the selected genes ranging from the hypothetical promoter area (approximately 10 kb upstream) until approximately 5 kb downstream of the gene. SNP codes, locations, and frequencies were obtained from NCBI (www.ncbi.nlm.nih.gov/SNP), HapMap (www.hapmap.org), and Illumina databases. A minor allele frequency (MAF) threshold of 0.05 in the HapMap CEU population and an ‘Illumina score’ not lower than 0.6 (as recommended by manufacturer) were established to ensure high genotyping success rate of the SNPs selected.

SNP genotyping was done using the Golden Gate Assay according to manufacturer’s protocol (Illumina, San Diego, CA, USA), as previously described [30].

### Statistical analysis

Quality control processes and allelic and genotypic association tests were performed using the SNPator software (www.snpator.com). Additional statistical analyses and plots were conducted using the R statistical framework. All genetic analyses were performed estimating the effect of the minor allele in the Spanish population.

For all polymorphisms studied, Fisher’s exact test was used both to test for deviations from Hardy-Weinberg equilibrium (HWE) between sexes and to compare allele counts between female and male individuals. Bonferroni correction was applied and *P* values higher than 1.37 × 10^−4^ were considered in HWE.

Associations between the genotyped SNPs and various pigmentation and sun sensitivity traits were assessed via logistic regression, coded additively for each copy of the minor allele. This was done for males and females separately, with eye colour (blue/green versus brown/black), hair colour (brown/black versus blond/red), skin colour (fair versus dark), number of naevi (≥50 versus <50), presence of lentigines (yes versus no), and childhood sunburn (yes versus no) as the outcome variables. Genotype-related Odds Ratios (ORs), their corresponding 95 % confidence intervals (CIs) and associated *P* values were estimated. Results of the association analysis were represented using volcano plots, mapping significance (−log10 *P* value) versus fold-change (log2 OR) for the comparison between individuals for eye colour, hair colour, skin colour, presence of lentigines, childhood sunburns and naevi number separately. *P* values were two sided and those lower than 0.01 were considered statistically significant (since six pigmentation traits were studied separately, statistical significant threshold of *P* value < 0.05/6 = 0.01).

In order to have an overview of all the significant estimates obtained in the sex-specific logistic regression analyses, we evaluated the differences in the number of polymorphisms associated both with protective and risk phenotypes between sex groups (*P* values < 0.05), using 2 × 2 contingency tables and performing a Fisher’s exact test.

Logistic regression was performed to re-assess associations between genotypes and melanoma risk done previously [30], but separating individuals by sex in order to estimate sex-specific ORs, 95 % CIs and *P* values. As mentioned above, the minor allele was also modelled under an additive model. Using the same criteria as in the analysis of pigmentation traits, two-sided *P* values lower than 0.01 were considered to constitute evidence of association.

Finally, we performed a sex-differentiated regression estimate test for each SNP for all phenotypic traits. We tested for equality of sex-specific allelic effects with the aim of obtaining sex-differentiated *P* values [31], and a statistical significance threshold of sex-differentiated *P* value < 0.05 was used. Briefly, for each sex-specific association test, sex-specific beta coefficients (log ORs) and the corresponding standard errors were evaluated using a Chi-square test with one degree of freedom. Then, a Chi-square test with two degrees of freedom was performed for each SNP, in which the previously calculated female-specific and male-specific Chi-square statistics were added up. Finally, a test of heterogeneity of allelic effects between males and females using a Chi-square test with one degree of freedom was conducted. A significant sex-specific and sex-differentiated *P* value is required to verify a potential significance in allelic effect by sex, following the same criteria used by Kocarnik and cols. [32]. Manhattan plots were used to display the strength of significant differences between male-only and female-only associated effects (−log10 sex-differentiated *P* value) for each trait tested.

## Results and discussion

Our sample set included 599 females and 458 males of Spanish ancestry. Median age was 45 years (range 18–92) for females and 47 years (range 18–92) for males. Regarding control individuals, mean age was 42 years (range 18–91) for females and 45 years (range 18–90) for males. Melanoma cases presented a median age of 52 years (range 18–92) for females and 53 years (range 18–92) for males. Since age differences were not observed between sample subsets (*P* value > 0.05), association analyses were not adjusted by age.

From an initial list of 384 tag-SNPs selected, 21 SNPs (5.4 %) were discarded due to failed genotyping (no PCR amplification, insufficient intensity for cluster separation or poor cluster definition). All 363 remaining SNPs were in HWE after Bonferroni correction (Additional file 1: Table S1). Minor allele frequencies estimated for each SNP were almost identical for females and males, with a remarkable linear correlation (*R*^2^) of 0.982 (Additional file 1: Figure S1).

### Association with phenotypic characteristics by sex

In a previous study published by our group, the association of some genes with phenotypic characteristics was reported [30]. However, analyses were performed without taking into account sex data. In the current study, samples were additionally stratified by sex to evaluate differences in pigmentation and sun response between males and females.

Thirty four SNPs showed association with at least one pigmentation trait, and 42 SNPs were associated with at least one sun response trait studied (*P* < 0.01) (Additional file 1: Tables S2 and S3). Each of these polymorphisms displayed a moderate effect on pigmentation in our Spanish population dataset. Our results showed apparent differences in the direction of the association with the pigmentation characteristics, with variants showing ORs below 1.0 correlated with dark pigmentation and/or good tolerance to sunlight, and variants with ORs above 1.0 associated with light pigmentation and/or poor tolerance to sunlight. Variants in these genes most likely play important roles in the differences in pigmentation and tanning response among individuals of the Spanish population, and subsequently also in skin cancer risk [33].

**Fig. 1:**
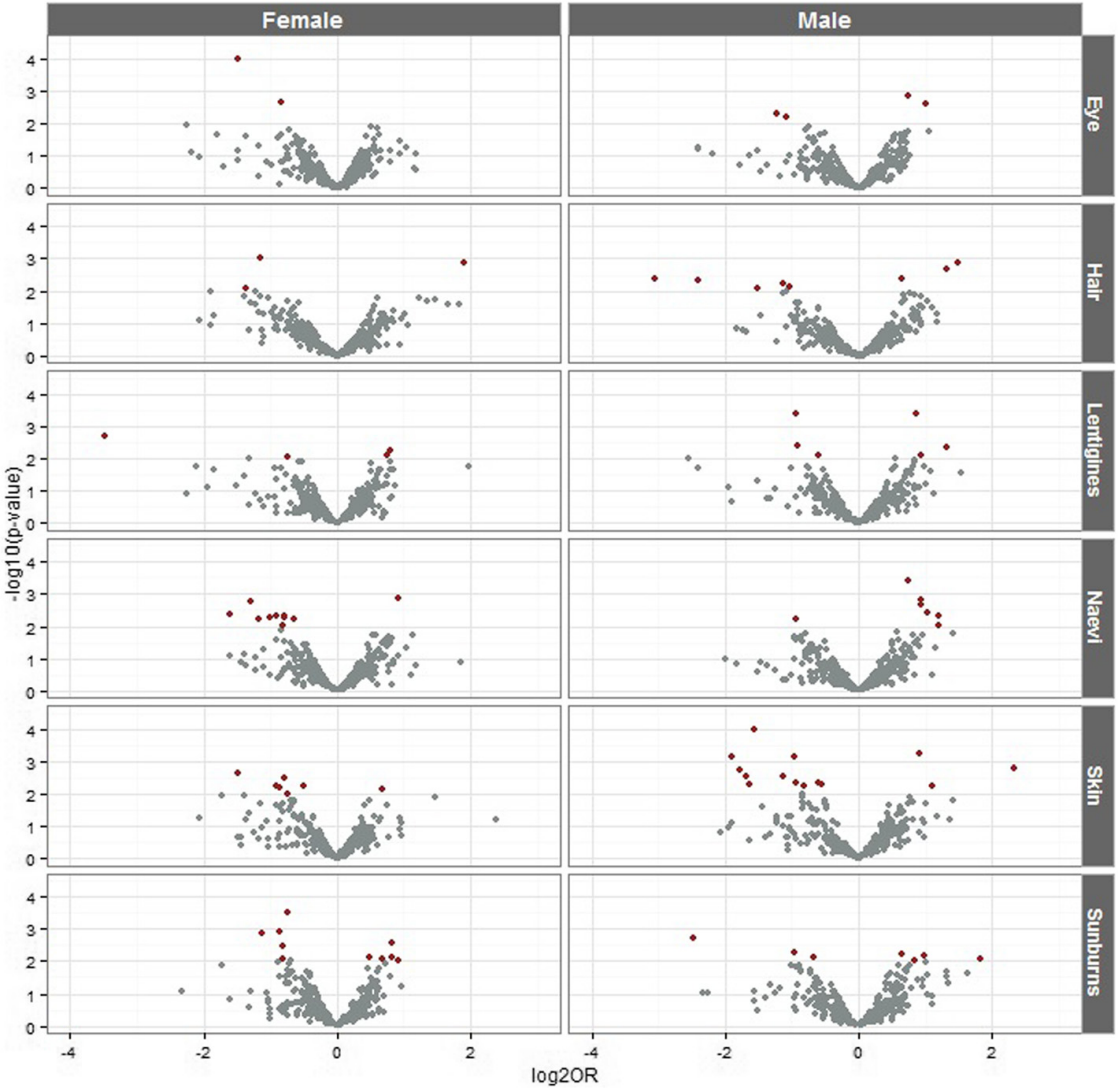
*Volcano plots* showing significance (−log10 *P* value) versus fold change (log2 OR) for pigmentation and sun sensitivity traits separated by sex. *Red dots* indicate SNPs with a significant fold change (*P* values < 0.01)

Representations of −log10 *P* values versus log2 ORs comparing 599 female individuals to 458 males for eye colour, hair colour, skin colour, presence of lentigines, childhood sunburns and naevi number are shown in Fig. 1. Detailed information on rs numbers, genes, chromosome locations, minor alleles, ORs, 95 % CIs, and *P* values for pigmentation and sun response characteristics are summarised in Additional file 1: Tables S2 and S3.

**Fig. 2:**
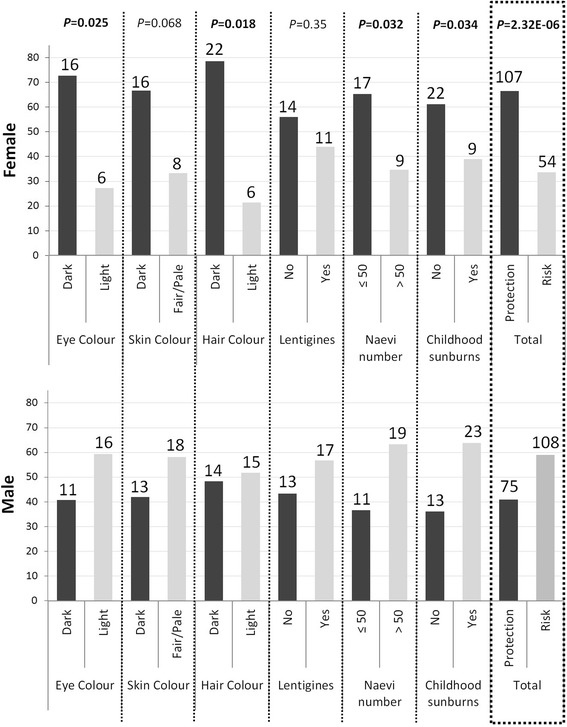
Distribution of the SNPs associated with pigmentation and sun sensitivity traits separated by sex. The percentage of each phenotype (protection or risk) is calculated taking into account the total number of significant SNPs associated in males and females (*P* values < 0.05). Percentages are represented by *bars* of the corresponding colour. The *number on the top of each bar* represents the count of associated SNPs in each category

Sex-specific analyses in this study showed significant differences in the pattern of association with pigmentation and tanning response traits between male and female individuals. Out of all SNPs with significant sex-specific associations, we found significantly more SNPs associated with dark pigmentation or sun protection in female than in male individuals (107 versus 75; *P* = 2.32 × 10^−6^), the latter being more commonly associated with light pigmentation and poor sun tolerance – traits highly associated with melanoma predisposition [9] (Fig. 2). The percentage of SNPs associated with both dark eye and dark hair colour in females was higher than in males (72.72 versus 40.74 %, *P* = 0.025; 78.57 versus 48.28 %, *P* = 0.018, respectively). This association pattern was also observed for skin colour, but without significance (66.67 versus 41.94 %, *P* = 0.068). In addition, female individuals presented more SNPs associated with both ≤50 naevi and absence of childhood sunburns than males (65.38 versus 36.67, *P* = 0.032; 61.11 versus 36.11 %, *P* = 0.034; respectively). On the other hand, a similar percentage of SNPs associated with absence of lentigines was observed in both female and male individuals (56.00 versus 43.33 %, *P* = 0.35). A representation of the distribution/count of polymorphisms associated with phenotype groups for each trait studied, separated by sex, is displayed in Fig. 2.

It is important to note that these associations do not reflect differences in the allelic frequencies of these pigmentation genes between males and females. These results basically indicate that, for a given genotype, the allelic effects on the phenotypic traits are shown to be significantly different in both sexes.

**Fig. 3:**
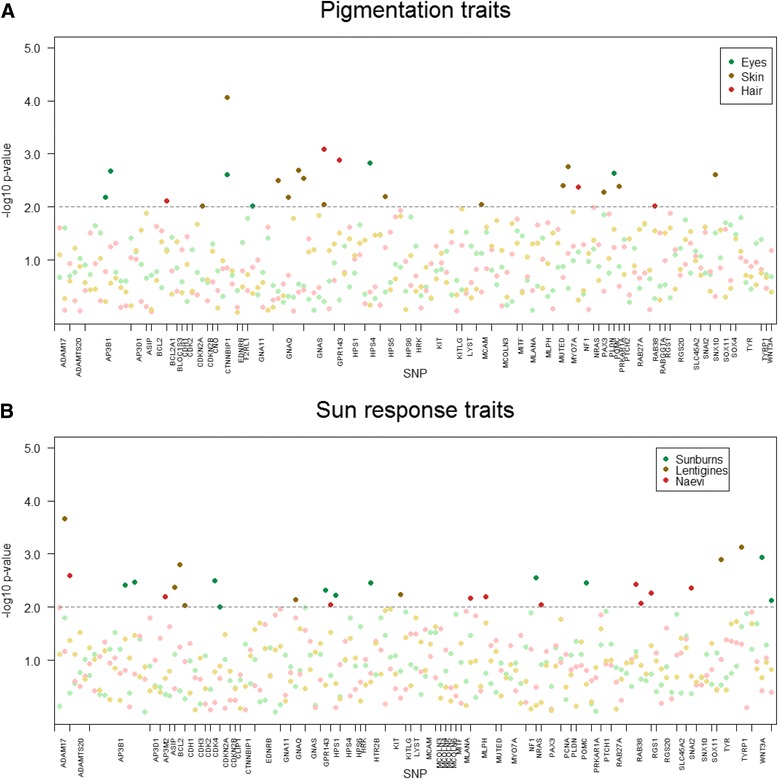
Manhattan plots displaying the strength of significant differences between male-only and female-only associated effects (−log10 sex-differentiated *P* value) for **a**) pigmentation and **b**) sun sensitivity traits. *Darker dots* of the corresponding colour indicate SNPs with a significant fold change (sex-differentiated *P* values < 0.01)

Additionally, sex-differentiated analysis was performed in order to test for equality between male-specific and female-specific regression estimates. Sex-differentiated *P* values are represented in Fig. 3. A significant sex-specific and sex-differentiated SNP association is required to establish a potential difference in effect for each polymorphism by sex. Three SNPs showed a strong potential sex-difference in eye colour effect, 10 SNPs in skin colour effect, 3 SNPs in hair colour effect, 4 SNPs in sunburns effect, 5 SNPs in lentigines effect, and 5 SNPs in naevi effect (*P* < 0.01). Among these SNPs, *PLDN* SNP rs12909221, *GPR143* SNP rs2521667, *POMC* SNP rs6734859, *AP3M2* SNP rs7009632, *BCL2* SNP rs1462129, and *TYRP1* SNP rs10809828 were associated with light pigmentation and poor sun tolerance in males. Only one polymorphism, rs2521578 on the *GPR143* gene, showed a high association with poor sun tolerance in females (Additional file 1: Tables S2 and S3).

Promising differences in allelic effect by sex were also observed for *TYR* SNP rs1042602. Females and males showed statistically significant effects in opposite directions for this SNP, and this difference in effect by sex would remain hard to discriminate from chance. Indeed, a sex-differentiated *P* value of 1.30 × 10^−3^ was estimated for rs1042602, as shown in Fig. 3.

Polymorphisms showing potential differences in allelic effect by sex are located on genes that have functions related to melanocyte development, melanosome formation, maturation, and transportation, as well as to skin cancer [6, 9, 10, 15, 16, 30, 34–37]. Interestingly, we also found associations between pigmentation phenotypes and several genes—*CDKN2A*, *GNA11*, *NRAS*, and *WNT3A*—involved in the up-regulation of melanogenic genes, the activation, survival, and proliferation of the melanocyte, and/or the processes leading to carcinogenesis.

### Associations with melanoma risk by sex

In a previous study published by our group, the association of 65 gene regions with melanoma risk was reported [30]. However, at that time, no sex stratification was applied to perform the association analysis. In this work, we have carried out an analysis of association between genotypes and melanoma risk for female and male individuals separately.

Sixteen SNPs located in 10 genes showed consistent male- or female-specific association with melanoma risk. Eleven of those SNPs showed potential differences in effect by sex, since *P* values obtained in the sex-differentiated regression estimate test were lower than 0.05. Detailed information on rs numbers, genes, chromosome locations, minor alleles, ORs, 95 % CIs, and *P* values for melanoma risk are summarised in Table 1.

**Table 1:** SNPs highly associated with melanoma risk in sex-stratified analysis

				Melanoma				
				Female		Male		Sex-diff
Gene	SNP ID	Chr	mA	*P* value	OR	*P* value	OR	*P* value
*AP3B1*	rs11742673	5	A	*0.0020*	*1.43 (1.14–1.80)*	0.88	0.98 (0.75–1.28)	*0.0210*
*CDK2/SILV*	rs2069398	12	A	*3.03E−4*	*0.47 (0.31–0.71)*	0.06	0.60 (0.35–1.03)	*0.0016*
*F2RL1*	rs2242991	5	G	0.56	0.92 (0.68–1.22)	*0.0076*	*1.61 (1.13–2.29)*	*0.0067*
*GPR143*	rs2521667	X	G	0.46	0.89 (0.67–1.20)	*7.04E−4*	*1.89 (1.30–2.74)*	*7.85E−4*
	rs2732872	X	C	0.78	0.96 (0.73–1.27)	*8.43E−4*	*1.81 (1.27–2.57)*	*0.0052*
*KIT*	rs6554198	4	G	0.46	0.92 (0.73–1.15)	*0.0027*	*0.67 (0.51–0.87)*	0.07
*MYO7A*	rs3758708	11	A	0.20	1.30 (0.87–1.95)	*4.12E−4*	*2.38 (1.46–3.90)*	*0.0480*
*RAB38*	rs524121	11	C	*0.0086*	*0.51 (0.30–0.85)*	0.36	1.32 (0.73–2.38)	*0.0210*
*RGS20*	rs6981243	8	C	0.38	0.90 (0.71–1.14)	*0.0021*	*0.66 (0.51–0.86)*	*0.0490*
*SLC45A2*	rs35414	5	T	0.03	0.77 (0.61–0.97)	*0.0018*	*0.66 (0.51–0.85)*	0.39
	rs35415	5	A	0.03	0.78 (0.62–0.97)	*0.0080*	*0.70 (0.54–0.91)*	0.35
*TYR*	rs1042602	11	A	0.35	1.11 (0.89–1.40)	*0.0047*	*0.68 (0.53–0.89)*	*0.0081*
	rs12270717	11	C	0.60	1.07 (0.82–1.40)	*0.0032*	*1.56 (1.16–2.09)*	0.09
	rs17793678	11	T	0.32	1.14 (0.88–1.49)	*0.0036*	*1.55 (1.15–2.08)*	0.26
	rs2186640	11	G	0.39	0.90 (0.71–1.15)	*9.20E−4*	*1.57 (1.20–2.05)*	*0.0380*
	rs5021654	11	C	0.47	0.92 (0.72–1.16)	*2.03E−4*	*1.66 (1.27–2.17)*	*0.0014*

Entries in italics indicate statistically significant results

*SNP* single nucleotide polymorphism, *Chr* chromosome, *mA* minor allele, *OR* odds ratio per minor allele, *CI* confidence interval, *Sex-diff* sex-differentiated meta-regression estimate test

Among these 11 SNPs, we found six SNPs located in 4 genes showing a strong difference in melanoma risk effect when samples were stratified by sex – sex-specific and sex-differentiated *P* values lower than 0.01. *F2RL1* SNP rs2242991, *GPR143* SNPs rs2521667, and rs2732872, and *TYR* SNP rs5021654 increased melanoma predisposition in males as opposed to females. Additionally, a strong melanoma protective effect was displayed by rs2069398 on *CDK2/SILV* in females only. These SNPs were also associated with pigmentation and sun tolerance in opposite directions in males (ORs > 1, melanoma risk traits) versus females (ORs < 1, melanoma protective traits), supporting lower melanoma predisposition and incidence in females than in males. Oppositely, rs1042602 on the *TYR* gene showed a melanoma protective effect in males compared to females. Therefore, these results are in accordance with the association between rs1042602 and dark pigmentation and good sun tolerance in males but not in females (Additional file 1: Tables S2 and S3).

These genes with potential differences in melanoma risk effect by sex are graphically represented in Additional file 1: Figure S2. The *F2RL1/PAR2* gene, expressed in keratinocytes but not in melanocytes, is a G-protein coupled receptor involved in melanosome transfer [38], and changes in its expression pattern are correlated with skin cancer progression [39]. The *GPR143* gene, located in the X chromosome, encodes for a G-protein coupled receptor for tyrosine, L-DOPA, and dopamine localised on melanosomal membranes and plays an important role in melanosome biogenesis, organisation and transport. Ocular albinism type 1 (OA1; MIM300500) is caused by mutations in *GPR143* and is transmitted as an X-linked trait. The *TYR* gene codes for another melanosomal membrane-bound enzyme involved in the rate-limiting steps of melanogenesis. Mutations in the *TYR* gene are associated with light pigmentation, freckling and sun sensitivity—well-recognised melanoma risk factors—as well as with melanoma [30, 40]. The *CDK2* gene, which overlaps with the melanocyte-specific gene *SILV*, is also important for melanoma growth and proliferation [41]. SILV melanosomal matrix protein represents a melanoma-specific antigen recognised by tumour infiltrating cytotoxic T lymphocytes [42].

A recent study is worth mentioning in this respect. According to Kocarnik and cols. (2014), *SLC45A2* SNP rs16891982, the non-synonymous mutation F374L located in exon 5, influenced melanoma risk differently by sex, with higher melanoma risk for males than females, probably through alterations in melanogenesis and pigmentation [32]. In our study, two SNPs on the *SLC45A2* gene (rs35414 and rs35415) displayed associations with melanoma in both female-only and male-only analysis, although they do not present significant sex-differentiated *P* values. It is important to note that the minor allele of these two SNPs showed a protective effect for melanoma and that the allele frequencies for these protective minor alleles, causing a darker pigmentation, are actually quite common in the Spanish population, as opposed to Northern European populations. Subsequently, this association was stronger in males (rs35414: OR = 0.66, 95 % CI 0.51–0.85, *P* = 0.0018; and rs35415: OR = 0.70, 95 % CI 0.54–0.91, *P* = 0.008) than in females (rs35414: OR = 0.77, 95 % CI 0.61–0.97, *P* = 0.026; and rs35415: OR = 0.78, 95 % CI 0.62–0.97, *P* = 0.029). In the male-only but not in the female-only analysis, rs35414 and rs35415 tended to be also associated with dark pigmentation and the absence of childhood sunburns (*P* < 0.05). It is important to state here that in the work by Kocarnik and cols., it was the major allele—in the Caucasian population—of the *SLC45A2* SNP that was in fact modelled as the purposed risk allele for melanoma, while in this study it is the minor allele of the two *SLC45A2* SNPs that was actually used as reference to perform the analyses. Therefore, the genetic effect shown by the *SLC45A2* gene in our study exhibits the opposite direction that the one displayed by the Kocarnik and cols. work.

We are aware of the limitations of the current work. Firstly, the sample size was relatively restricted after dividing by sex the complete sample set, probably resulting in limited statistical power to detect modest effects for additional SNPs. Unfortunately, there are not previously published genome-wide studies presenting data stratified by sex, hindering chances of enlarging the sample size. Secondly, the subjective nature of the attributes considered may be another reason for misclassification. Thirdly, we presented two-sided unadjusted *P* values for the associations considered; and the level of statistical significance considered was lower than the threshold required to declare unequivocally positive results. However, the results of this work—as well as other previous studies [24, 25, 32]—show that there is a strong tendency showing sex-differentiated genetic effects in pigmentary traits. Therefore, we believe that the work presented here is nonetheless reporting very interesting findings. For all these limitations, replication of our findings is essential before venturing on drawing firmer conclusions.

The results of this study suggest that there are indeed sex-specific genetic effects in human pigmentation, with larger effects for darker pigmentation in females compared to males. A plausible cause might be the differentially expressed melanogenic genes in females due to higher oestrogen levels. These sex-specific genetic effects would help explain the presence of darker eye and skin pigmentation in females, as well as the well-known higher melanoma risk displayed by males.

## Conclusions

Overall, the results of this work reveal the presence of sex-specific effects in human pigmentation that might be important not only in skin colour and sensitivity to sunlight but also in the higher incidence of melanoma described in males. These findings also show that, at times, sex-stratified analyses enrich genetic association studies with valuable information and knowledge.

## Additional file

Additional file 1: This contains **Tables S1–S3** and **Figures S1–S2.** **Table S1.** List of 363 successfully genotyped SNPs, Minor allele frequencies for all samples, males and females, and HWE *P* value. **Table S2.** List of SNPs associated with pigmentation traits in females and males. **Table S3.** List of SNPs associated with sun response traits in females and males. **Figure S1.** Comparison of minor allele frequencies, female versus male individuals. **Figure S2.** A selection of genetic factors affecting pigmentation and sun sensitivity in humans. (PDF 417 kb)

## Abbreviations

CEU: Utah residents with Northern and Western European ancestry
Chr: chromosome
CI: confidence interval
HWE: Hardy-Weinberg equilibrium
mA: minor allele
MAF: minor allele frequency
OR: odds ratio
*P*: *P* value
Sex-diff: sex-differentiated regression estimates test
SNP: single nucleotide polymorphism
UV: ultraviolet
