# Supplementary material for: Sex-specific genetic effects associated with pigmentation, sensitivity to sunlight, and melanoma in a population of Spanish origin
Source: Biol Sex Differ. 2016 Mar 18;7:17. doi: 10.1186/s13293-016-0070-1 (PMC4797181; doi:10.1186/s13293-016-0070-1)
Supplement: Additional file 1: — This contains Tables S1–S3 and Figures S1–S2. Table S1. List of 363 successfully genotyped SNPs, Minor allele frequencies for all samples, males and females, and HWE P value. Table S2. List of SNPs associated with pigmentation traits in females and males. Table S3. List of SNPs associated with sun response traits in females and males. Figure S1. Comparison of minor allele frequencies, female versus male individuals. Figure S2. A selection of genetic factors affecting pigmentation and sun sensitivity in humans. (PDF 417 kb) [file 13293_2016_70_MOESM1_ESM.pdf]

SUPPLEMENTARY MATERIAL

Table S1. List of 363 successfully genotyped SNPs, Minor allele frequencies for all samples, males and females, and HWE *p*-value 2

Table S2. List of SNPs associated with pigmentation traits in females and males 9

Table S3. List of SNPs associated with sun response traits in females and males 12

Figure S1. Comparison of minor allele frequencies, female versus male individuals 15

Figure S2. A selection of genetic factors affecting pigmentation and sun sensitivity in humans 16

Table S1. List of 363 successfully genotyped SNPs, Minor allele frequencies for all samples, males and females, and HWE p-values

					All samples			Males		Females	
Gene	SNP rs#	Chr	Position on Chr (bp)	Location in Gene	mA	MAF	HWE p-value	MAF	HWE p-value	MAF	HWE p-value
**ADAM17**	rs12473402	2	9550169	intron	C	0.370	0.34	0.374	0.28	0.366	0.97
**ADAM17**	rs12475630	2	9551832	intron	T	0.325	0.61	0.327	0.49	0.327	0.18
**ADAM17**	rs12992105	2	9581324	intron	C	0.165	0.13	0.169	0.84	0.162	0.09
**ADAM17**	rs17524425	2	9543455	3' downstream	G	0.213	0.76	0.202	0.78	0.221	0.74
**ADAM17**	rs4258773	2	9588663	intron	G	0.462	0.82	0.471	0.34	0.453	0.44
**ADAMTS20**	rs1510521	12	42193473	intron	G	0.342	0.90	0.321	0.72	0.356	0.82
**ADAMTS20**	rs17093450	12	42221612	intron	A	0.121	0.76	0.120	0.42	0.121	0.74
**ADAMTS20**	rs2048348	12	42215388	intron	A	0.105	0.89	0.123	0.98	0.092	0.81
**ADAMTS20**	rs2062731	12	42227223	intron	C	0.091	0.86	0.094	0.63	0.087	0.57
**ADAMTS20**	rs275630	12	42227033	intron	T	0.305	0.43	0.294	0.37	0.310	0.57
**ADAMTS20**	rs3764467	12	42105989	intron	T	0.093	0.94	0.084	0.46	0.099	0.68
**ADAMTS20**	rs7297057	12	42117952	intron	T	0.187	0.34	0.207	0.91	0.177	0.16
**ADAMTS20**	rs7960952	12	42212399	intron	C	0.420	0.94	0.407	0.88	0.431	0.61
**ADAMTS20**	rs9634256	12	42226764	intron	G	0.432	0.40	0.435	0.07	0.431	0.76
**AP3B1**	rs10514134	5	77355203	intron	A	0.094	0.96	0.098	0.93	0.090	0.79
**AP3B1**	rs10805919	5	77628680	5' upstream	C	0.237	0.72	0.258	0.27	0.218	0.69
**AP3B1**	rs11742673	5	77535322	intron	A	0.435	0.54	0.419	0.20	0.452	0.76
**AP3B1**	rs11746090	5	77375602	intron	A	0.249	0.91	0.230	0.97	0.261	0.91
**AP3B1**	rs12657894	5	77537609	intron	A	0.372	0.76	0.379	0.33	0.361	0.29
**AP3B1**	rs13172957	5	77611135	intron	G	0.329	0.72	0.330	0.22	0.324	0.56
**AP3B1**	rs17191796	5	77444232	intron	G	0.109	0.76	0.112	0.69	0.105	0.94
**AP3B1**	rs2636986	5	77421437	intron	T	0.215	0.41	0.223	0.81	0.210	0.34
**AP3B1**	rs34436	5	77394844	intron	G	0.106	0.97	0.109	0.88	0.107	0.70
**AP3B1**	rs389110	5	77408005	intron	A	0.215	0.36	0.223	0.81	0.211	0.28
**AP3B1**	rs4703747	5	77340718	intron	G	0.191	0.91	0.197	0.88	0.185	0.95
**AP3B1**	rs6453373	5	77460784	coding exon	A	0.075	0.61	0.073	0.88	0.077	0.73
**AP3B1**	rs6453374	5	77543915	intron	A	0.290	0.82	0.282	0.42	0.294	0.60
**AP3D1**	rs10413398	19	2057696	intron	C	0.201	0.43	0.204	0.42	0.197	0.75
**AP3D1**	rs17604954	19	2062382	intron	A	0.117	0.63	0.124	0.25	0.113	0.60
**AP3D1**	rs2238599	19	2091009	intron	C	0.460	0.65	0.474	0.29	0.449	0.16
**AP3D1**	rs2240655	19	2066790	intron	T	0.132	**0.05**	0.134	0.36	0.131	0.07
**AP3D1**	rs3786971	19	2092209	intron	T	0.438	0.19	0.428	0.91	0.447	0.15
**AP3D1**	rs7256735	19	2120121	5' upstream	G	0.086	0.89	0.088	0.70	0.088	0.72
**AP3M2**	rs2070713	8	42164812	3' downstream	T	0.417	0.61	0.423	0.45	0.408	0.93
**AP3M2**	rs7009632	8	42127343	5' upstream	G	0.422	0.14	0.423	0.27	0.421	0.19
**AP3M2**	rs7823824	8	42117935	5' upstream	A	0.433	0.48	0.447	0.24	0.424	0.75
**AP3M2**	rs8178890	8	42154511	3' downstream	T	0.082	0.91	0.071	0.71	0.088	0.59
**ASIP**	rs6142129	20	32283532	5' upstream	G	0.334	0.46	0.324	0.91	0.338	0.62
**ASIP**	rs819133	20	32333975	intron	T	0.126	0.60	0.135	0.69	0.124	0.73
**BCL2**	rs1462129	18	59131851	intron	T	0.467	0.91	0.480	0.27	0.460	0.19
**BCL2**	rs1564483	18	58945634	3' UTR	T	0.253	0.86	0.154	0.74	0.241	0.93
**BCL2**	rs4987852	18	58944901	3' UTR	C	0.069	0.74	0.057	0.88	0.077	0.56
**BCL2**	rs4987853	18	58944635	3' UTR	C	0.256	0.43	0.264	0.54	0.245	0.72
**BCL2**	rs949037	18	59129993	intron	A	0.437	0.59	0.445	0.76	0.432	0.15
**BCL2A1**	rs11636338	15	78032645	3' downstream	C	0.337	**0.03**	0.344	0.94	0.331	**0.03**
**BCL2A1**	rs17215263	15	78043311	intron	G	0.314	0.26	0.323	0.89	0.303	0.14
**BCL2A1**	rs6495460	15	78044410	intron	C	0.128	0.57	0.141	0.37	0.120	0.93
**BLOC1S3**	rs7253652	19	50372974	5' upstream	C	0.097	0.45	0.106	0.48	0.089	0.78
**CDH1**	rs11075699	16	67360288	intron	G	0.419	0.79	0.398	0.45	0.438	0.44
**CDH1**	rs16260	16	67328535	5' upstream	A	0.296	0.56	0.302	0.75	0.290	0.62
**CDH1**	rs1801552	16	67414942	coding exon	T	0.369	0.77	0.320	0.15	0.411	0.79
**CDH1**	rs2276329	16	67420815	intron	C	0.055	0.60	0.066	0.25	0.047	0.71
**CDH1**	rs2902186	16	67319398	5' upstream	G	0.097	0.55	0.110	0.74	0.090	0.25

**CDH1**	rs7188750	16	67400396	intron	A	0.153	0.73	0.168	0.97	0.142	0.69
**CDH1**	rs7203904	16	67420442	intron	C	0.218	0.53	0.242	0.23	0.200	**0.04**
**CDH1**	rs8059139	16	67402468	intron	G	0.061	0.85	0.068	0.98	0.056	0.76
**CDH1**	rs8061932	16	67420098	intron	C	0.174	0.27	0.197	0.96	0.158	0.10
**CDH3**	rs11075692	16	67239293	intron	G	0.378	0.35	0.370	0.30	0.387	0.69
**CDH3**	rs1124770	16	67263914	intron	G	0.150	0.54	0.153	0.93	0.146	0.49
**CDH3**	rs1886700	16	67243406	intron	T	0.128	0.37	0.130	0.70	0.127	0.14
**CDH3**	rs3114398	16	67264434	intron	G	0.283	0.69	0.291	0.96	0.277	0.71
**CDH3**	rs3118230	16	67266860	intron	T	0.093	0.46	0.103	0.97	0.087	0.32
**CDK4**	rs2069502	12	56430932	intron	T	0.237	0.85	0.253	0.42	0.235	0.73
**CDK4**	rs2270777	12	56431423	non-coding exon	C	0.469	0.97	0.490	0.33	0.459	0.51
**CDKN2A**	rs11515	9	21958199	3' UTR	G	0.170	0.46	0.170	0.81	0.170	0.30
**CDKN2A**	rs2518719	9	21960427	intron	G	0.158	0.43	0.175	0.84	0.146	0.53
**CDKN2A**	rs3731239	9	21964218	intron	G	0.317	0.21	0.308	0.55	0.324	0.36
**CDKN2A**	rs3731257	9	21956221	3' downstream	A	0.299	0.57	0.294	0.38	0.299	0.85
**CDKN2B**	rs1063192	9	21993367	3' UTR	G	0.357	0.20	0.365	0.50	0.354	0.20
**CDKN2B**	rs2811712	9	21988035	3' downstream	G	0.117	0.45	0.122	0.96	0.114	0.40
**CDKN2B**	rs3218009	9	21988757	3' downstream	C	0.085	0.88	0.077	0.93	0.087	0.73
**CDKN2B**	rs3218020	9	21987872	5' upstream	A	0.442	0.77	0.429	0.97	0.446	0.52
**CDKN2B**	rs495490	9	22000412	5' upstream	G	0.108	0.85	0.115	0.63	0.105	0.48
**CLIP1**	rs7388	12	121322137	intron	A	0.225	0.93	0.226	0.96	0.224	0.73
**CNO**	rs10937751	4	6762957	5' upstream	A	0.287	0.28	0.282	0.42	0.289	0.43
**CNO**	rs3172604	4	6769676	3' UTR	G	0.355	0.57	0.353	0.78	0.351	0.57
**CNO**	rs4689527	4	6771878	3' downstream	G	0.366	0.20	0.360	1.00	0.365	0.09
**CTNNBIP1**	rs11828	1	9909594	3' downstream	C	0.187	0.32	0.186	0.40	0.185	0.54
**CTNNBIP1**	rs12128766	1	9859628	intron	C	0.423	0.60	0.399	0.76	0.435	0.44
**CTNNBIP1**	rs1220392	1	9829390	3' downstream	A	0.150	0.24	0.155	0.99	0.144	0.15
**CTNNBIP1**	rs2379107	1	9833878	intron	G	0.167	0.63	0.149	0.41	0.179	0.83
**CTNNBIP1**	rs4846104	1	9900834	intron	G	0.064	0.78	0.060	0.92	0.063	0.69
**DCT**	rs2031527	13	93921918	intron	T	0.194	0.20	0.207	0.28	0.186	0.56
**DCT**	rs3782972	13	93901047	intron	C	0.165	0.21	0.163	0.70	0.167	0.13
**DCT**	rs3782973	13	93900698	intron	G	0.165	0.21	0.162	0.82	0.169	0.10
**DCT**	rs9301959	13	93908106	intron	C	0.451	0.55	0.455	0.90	0.447	0.32
**DCT**	rs9516418	13	93909510	intron	C	0.374	0.39	0.384	0.29	0.368	0.71
**DCT**	rs9524493	13	93899790	intron	G	0.460	0.07	0.451	0.12	0.464	0.39
**EDNRB**	rs11149080	13	77365761	3' downstream	G	0.424	0.45	0.425	0.86	0.421	0.30
**EDNRB**	rs3027110	13	77377230	intron	C	0.072	0.67	0.075	0.88	0.071	0.47
**EDNRB**	rs3027129	13	77384402	intron	T	0.060	0.84	0.051	0.96	0.068	0.77
**EDNRB**	rs3818416	13	77372469	intron	A	0.281	0.58	0.298	0.61	0.267	0.81
**EDNRB**	rs4885491	13	77368351	3' UTR	A	0.160	0.48	0.165	0.62	0.157	0.61
**F2RL1**	rs2242991	5	76150615	5' UTR	G	0.183	0.13	0.171	0.16	0.191	0.33
**F2RL1**	rs2243004	5	76152873	intron	G	0.174	0.80	0.177	0.74	0.172	0.89
**F2RL1**	rs2243010	5	76153524	intron	T	0.158	0.48	0.166	0.60	0.151	0.39
**F2RL1**	rs2243051	5	76161558	intron	G	0.449	0.38	0.459	0.96	0.441	0.19
**F2RL1**	rs639342	5	76148625	5' upstream	A	0.165	0.71	0.163	0.97	0.168	0.73
**GNA11**	rs10407783	19	3066124	intron	T	0.345	0.13	0.347	0.69	0.339	0.10
**GNA11**	rs1104737	19	3047788	intron	T	0.385	0.10	0.376	**0.04**	0.389	0.66
**GNA11**	rs2238625	19	3055652	intron	C	0.456	0.97	0.456	0.41	0.452	0.50
**GNA11**	rs308039	19	3046927	intron	T	0.175	0.54	0.178	0.77	0.173	0.27
**GNA11**	rs308054	19	3056987	intron	A	0.429	0.11	0.427	0.13	0.435	0.37
**GNA11**	rs3746069	19	3065864	intron	T	0.161	0.90	0.158	0.96	0.164	0.85
**GNA11**	rs3786947	19	3065229	intron	A	0.389	0.21	0.392	0.37	0.383	0.31
**GNA11**	rs404632	19	3065545	intron	T	0.122	0.25	0.120	0.57	0.123	0.23
**GNA11**	rs4806907	19	3044163	5' upstream	C	0.393	0.90	0.379	0.23	0.404	0.39
**GNAQ**	rs10781468	9	79690225	intron	T	0.256	0.15	0.249	0.59	0.261	0.15
**GNAQ**	rs10869977	9	79647096	intron	A	0.443	0.19	0.447	0.42	0.439	0.33
**GNAQ**	rs11145647	9	79801980	intron	C	0.201	0.69	0.204	0.69	0.200	0.50
**GNAQ**	rs12686139	9	79681047	intron	C	0.463	0.81	0.466	0.57	0.462	0.79

**GNAQ**	rs1328533	9	79674282	intron	G	0.341	0.51	0.340	0.77	0.342	0.58
**GNAQ**	rs1328534	9	79674191	intron	T	0.242	0.80	0.239	0.44	0.245	0.68
**GNAQ**	rs1410552	9	79691494	intron	T	0.291	0.34	0.289	0.24	0.294	0.79
**GNAQ**	rs17724885	9	79659385	intron	G	0.069	0.98	0.068	0.80	0.070	0.82
**GNAQ**	rs17786974	9	79733027	intron	T	0.083	0.80	0.086	0.93	0.081	0.83
**GNAQ**	rs2296937	9	79696407	intron	C	0.120	0.98	0.122	0.53	0.119	0.75
**GNAQ**	rs3780302	9	79576374	intron	T	0.121	0.88	0.129	0.89	0.117	0.87
**GNAQ**	rs3858119	9	79524896	3' UTR	C	0.300	0.06	0.303	0.14	0.296	0.35
**GNAQ**	rs4237275	9	79724578	intron	C	0.254	0.49	0.262	0.16	0.251	0.80
**GNAQ**	rs4745672	9	79688048	intron	T	0.489	0.39	0.491	0.37	0.486	0.82
**GNAQ**	rs7028873	9	79717352	intron	T	0.290	0.33	0.288	0.13	0.292	0.99
**GNAS**	rs12625436	20	56878608	intron	A	0.424	0.58	0.433	**0.05**	0.420	0.33
**GNAS**	rs13831	20	56908586	3' UTR	A	0.254	0.18	0.246	0.29	0.261	0.38
**GNAS**	rs2145288	20	56877604	intron	C	0.297	0.31	0.293	0.44	0.303	0.09
**GNAS**	rs234623	20	56922359	3' downstream	A	0.478	0.14	0.483	0.87	0.473	0.06
**GNAS**	rs234627	20	56920375	3' downstream	T	0.266	0.25	0.277	0.12	0.255	0.99
**GNAS**	rs6026561	20	56860527	5' upstream	C	0.386	0.42	0.401	0.21	0.377	0.99
**GNAS**	rs6026574	20	56889890	intron	T	0.304	0.55	0.289	0.57	0.318	0.66
**GNAS**	rs6064714	20	56847535	5' upstream	G	0.131	0.19	0.139	0.97	0.127	0.10
**GNAS**	rs6092704	20	56901873	intron	C	0.086	0.86	0.083	0.57	0.087	0.74
**GNAS**	rs6100269	20	56912671	intron	T	0.107	0.94	0.104	0.99	0.108	0.84
**GNAS**	rs6123832	20	56851466	intron	T	0.422	0.70	0.425	0.12	0.421	0.51
**GNAS**	rs919197	20	56914328	intron	T	0.468	0.28	0.477	0.43	0.461	**0.05**
**GNAS**	rs965808	20	56841821	5' upstream	C	0.255	0.27	0.247	0.73	0.263	0.15
**GPR143**	rs11095519	X	9667373	intron	A	0.141	***	0.141	***	0.140	0.52
**GPR143**	rs2521578	X	9655630	intron	T	0.135	***	0.165	***	0.112	0.92
**GPR143**	rs2521667	X	9690838	intron	G	0.167	***	0.155	***	0.179	0.22
**GPR143**	rs2732872	X	9688521	intron	C	0.197	***	0.179	***	0.213	0.86
**GPR143**	rs5979160	X	9650197	3' downstream	C	0.168	***	0.163	***	0.164	0.72
**GPR143**	rs6654731	X	9666637	intron	C	0.047	***	0.044	***	0.044	0.96
**HPS1**	rs1061135	10	100179128	intron	G	0.488	0.21	0.485	0.72	0.461	0.32
**HPS1**	rs10786422	10	100194717	intron	T	0.276	0.35	0.287	0.90	0.270	0.27
**HPS1**	rs10883094	10	100178096	intron	A	0.208	0.46	0.219	0.42	0.204	0.72
**HPS1**	rs12242431	10	100179066	intron	C	0.113	0.66	0.103	0.78	0.122	0.83
**HPS1**	rs1739	10	100166329	3' UTR	A	0.315	0.08	0.319	0.15	0.316	0.22
**HPS1**	rs17535384	10	100184592	intron	G	0.140	0.83	0.133	1.00	0.143	0.67
**HPS1**	rs1886728	10	100183669	intron	C	0.444	0.46	0.439	0.49	0.448	0.53
**HPS1**	rs3750605	10	100165644	3' downstream	T	0.202	0.41	0.218	0.51	0.193	0.50
**HPS1**	rs7075080	10	100202364	intron	G	0.408	0.86	0.404	0.96	0.414	0.77
**HPS1**	rs7921146	10	100176591	intron	A	0.209	0.41	0.225	0.56	0.199	0.45
**HPS4**	rs16982145	22	25191721	intron	C	0.038	0.93	0.030	0.52	0.046	0.72
**HPS4**	rs17401652	22	25198490	intron	T	0.104	0.66	0.104	0.82	0.099	0.55
**HPS4**	rs1894707	22	25189086	intron	C	0.372	0.71	0.362	0.72	0.376	0.80
**HPS4**	rs3213583	22	25191545	intron	A	0.109	0.45	0.105	0.29	0.111	0.82
**HPS4**	rs3747129	22	25192041	coding exon	A	0.145	0.21	0.135	0.61	0.154	0.24
**HPS4**	rs9608491	22	25200985	intron	G	0.187	0.98	0.204	0.68	0.174	0.58
**HPS4**	rs9613187	22	25205370	intron	T	0.112	0.98	0.123	0.98	0.104	0.90
**HPS5**	rs12218	11	18247897	coding exon	C	0.483	0.62	0.495	0.67	0.465	0.81
**HPS5**	rs2049129	11	18261909	intron	C	0.165	0.60	0.162	0.84	0.166	0.64
**HPS5**	rs2305564	11	18274034	intron	A	0.496	0.37	0.481	0.34	0.480	0.68
**HPS5**	rs4353250	11	18280093	intron	T	0.309	0.44	0.300	0.82	0.311	0.46
**HPS5**	rs4757637	11	18264151	intron	C	0.363	0.58	0.347	0.62	0.373	0.50
**HPS5**	rs7131332	11	18252099	3' downstream	G	0.318	0.25	0.314	0.05	0.320	0.97
**HPS6**	rs3737243	10	103815737	coding exon	A	0.102	0.41	0.097	0.42	0.107	0.71
**HPS6**	rs3816	10	103817727	3' UTR	G	0.220	0.81	0.233	0.64	0.212	0.32
**HPS6**	rs6584475	10	103796783	intron	C	0.381	0.45	0.366	0.75	0.384	0.24
**HRK**	rs10507275	12	115778887	3' downstream	A	0.158	0.96	0.161	0.86	0.158	0.99
**HRK**	rs1112700	12	115790588	intron	A	0.139	0.92	0.143	0.71	0.138	0.79

**HRK**	rs4767462	12	115781956	3' UTR	G	0.065	0.95	0.076	0.66	0.060	0.62
**HRK**	rs884378	12	115792581	intron	A	0.261	0.57	0.270	0.96	0.258	0.47
**HTR2B**	rs10194776	2	231688263	intron	T	0.396	0.99	0.391	0.38	0.399	0.61
**HTR2B**	rs17619600	2	231684704	intron	C	0.090	0.72	0.093	0.98	0.087	0.74
**HTR2B**	rs2161891	2	231662335	intron	G	0.345	0.79	0.330	0.77	0.357	0.84
**HTR2B**	rs4973377	2	231690236	intron	A	0.172	0.43	0.173	0.92	0.175	0.30
**KIT**	rs1008658	4	55294193	intron	T	0.356	0.97	0.361	0.71	0.350	0.86
**KIT**	rs11735550	4	55283476	intron	C	0.134	0.28	0.129	0.43	0.136	0.55
**KIT**	rs13135792	4	55247087	intron	C	0.355	0.15	0.348	0.48	0.355	0.25
**KIT**	rs2213180	4	55296084	intron	G	0.184	0.78	0.166	0.94	0.198	0.82
**KIT**	rs2237025	4	55236636	intron	T	0.457	0.72	0.465	0.88	0.455	0.63
**KIT**	rs2298976	4	55283293	intron	G	0.128	0.89	0.144	0.75	0.112	0.92
**KIT**	rs4864920	4	55285378	intron	T	0.211	0.86	0.223	0.50	0.199	0.76
**KIT**	rs6554198	4	55216917	5' upstream	G	0.425	0.94	0.423	0.32	0.427	0.57
**KIT**	rs759083	4	55232238	intron	G	0.370	0.30	0.365	0.37	0.368	0.76
**KITLG**	rs10858753	12	87434601	intron	T	0.051	0.87	0.053	0.93	0.050	0.89
**KITLG**	rs10858758	12	87468649	intron	G	0.232	0.27	0.231	0.78	0.228	0.16
**KITLG**	rs11104903	12	87407546	3' downstream	G	0.131	0.49	0.130	0.95	0.128	0.36
**KITLG**	rs11610915	12	87426172	intron	A	0.014	0.94	0.016	0.95	0.013	0.99
**LYST**	rs11810173	1	233899178	intron	T	0.170	0.96	0.163	0.42	0.176	0.66
**LYST**	rs17714318	1	234105797	intron	A	0.071	0.92	0.069	0.74	0.073	0.85
**LYST**	rs3754230	1	233893206	intron	A	0.114	0.97	0.114	0.99	0.111	0.90
**LYST**	rs3768051	1	233926337	intron	T	0.127	0.95	0.110	0.29	0.138	0.52
**LYST**	rs6429238	1	233921363	intron	T	0.347	0.96	0.354	0.86	0.346	0.66
**LYST**	rs6699717	1	233888473	3' downstream	T	0.500	0.43	0.495	0.26	0.497	0.93
**LYST**	rs7541057	1	233926963	intron	C	0.491	0.78	0.498	0.45	0.487	0.79
**MCAM**	rs2249466	11	118686380	coding exon	T	0.314	0.35	0.327	0.35	0.308	0.81
**MCAM**	rs2511837	11	118684217	3' downstream	T	0.476	0.92	0.469	0.38	0.476	0.38
**MCAM**	rs6589732	11	118690557	intron	A	0.407	0.83	0.398	0.40	0.407	0.48
**MCOLN3**	rs10518327	1	85260108	intron	A	0.272	0.99	0.259	0.64	0.279	0.71
**MCOLN3**	rs10782537	1	85257061	3' UTR	C	0.218	0.54	0.223	0.78	0.220	0.50
**MCOLN3**	rs10873682	1	85270400	intron	A	0.434	0.44	0.429	0.68	0.438	0.57
**MCOLN3**	rs12030837	1	85291971	5' upstream	T	0.125	0.87	0.127	0.89	0.125	0.80
**MCOLN3**	rs12735211	1	85262088	intron	A	0.076	0.51	0.074	0.49	0.075	0.94
**MCOLN3**	rs2304641	1	85285986	intron	T	0.173	0.80	0.184	0.93	0.168	0.89
**MCOLN3**	rs6674050	1	85288762	5' upstream	C	0.222	0.29	0.213	0.53	0.224	0.39
**MCOLN3**	rs7522239	1	85256954	3' UTR	A	0.143	0.52	0.137	0.75	0.150	0.50
**MITF**	rs11128152	3	70049623	intron	T	0.185	0.55	0.188	0.53	0.186	0.89
**MITF**	rs13072665	3	70076003	intron	A	0.090	0.86	0.079	0.97	0.096	0.75
**MITF**	rs2131025	3	70098337	3' UTR	C	0.070	0.96	0.076	0.87	0.069	0.96
**MITF**	rs3821364	3	70094111	intron	C	0.246	0.36	0.266	0.30	0.235	0.75
**MITF**	rs7623610	3	70087971	intron	G	0.480	0.19	0.487	**0.04**	0.481	0.97
**MLANA**	rs1056796	9	5899152	3' UTR	T	0.310	0.72	0.306	0.87	0.320	0.70
**MLANA**	rs10758717	9	5874281	5' upstream	C	0.244	0.91	0.247	0.53	0.249	0.51
**MLANA**	rs10815300	9	5874564	5' upstream	T	0.177	0.96	0.180	0.96	0.180	0.96
**MLANA**	rs10815303	9	5891100	intron	T	0.115	0.59	0.118	0.53	0.115	0.81
**MLANA**	rs10815304	9	5898217	intron	C	0.221	0.95	0.217	0.93	0.227	0.99
**MLANA**	rs10975339	9	5885586	intron	T	0.327	0.54	0.316	0.95	0.334	0.36
**MLANA**	rs2150702	9	5883861	intron	G	0.461	0.89	0.457	0.27	0.466	0.72
**MLANA**	rs7872509	9	5893555	intron	C	0.186	0.98	0.189	0.74	0.187	0.86
**MLPH**	rs10173589	2	238239684	intron	G	0.159	0.48	0.161	0.28	0.152	0.97
**MLPH**	rs13011946	2	238225025	intron	T	0.048	0.92	0.050	0.96	0.047	0.93
**MLPH**	rs13383648	2	238229295	intron	C	0.122	0.78	0.115	0.82	0.128	0.67
**MLPH**	rs2292881	2	238216249	coding exon	T	0.087	0.84	0.082	0.98	0.091	0.66
**MLPH**	rs729389	2	238207118	intron	A	0.129	0.79	0.130	0.76	0.128	0.97
**MLPH**	rs7606177	2	238176913	5' upstream	C	0.237	0.62	0.242	0.29	0.235	0.88
**MLPH**	rs880931	2	238178741	intron	A	0.101	0.36	0.109	0.55	0.095	0.44
**MUTED**	rs2743989	6	8009034	intron	A	0.393	0.36	0.398	0.30	0.391	0.80

**MUTED**	rs2748376	6	7960128	3' UTR	T	0.409	0.85	0.409	0.96	0.415	0.77
**MUTED**	rs2815155	6	8010229	5' upstream	C	0.427	0.75	0.420	0.91	0.425	0.32
**MUTED**	rs3734591	6	7960820	3' UTR	C	0.171	0.70	0.177	0.45	0.173	0.73
**MUTED**	rs9328451	6	7963071	intron	T	0.190	0.96	0.192	0.72	0.190	0.80
**MYO7A**	rs11237123	11	76600594	coding exon	A	0.189	0.71	0.210	0.45	0.173	0.87
**MYO7A**	rs11605022	11	76550062	intron	G	0.424	0.62	0.432	0.87	0.419	0.30
**MYO7A**	rs12793189	11	76598738	intron	G	0.488	0.36	0.465	0.41	0.493	0.60
**MYO7A**	rs12793619	11	76599006	intron	A	0.264	0.56	0.286	0.96	0.248	0.60
**MYO7A**	rs3740760	11	76531649	intron	C	0.252	0.94	0.260	0.85	0.246	0.98
**MYO7A**	rs3740763	11	76551268	intron	T	0.422	0.80	0.420	0.76	0.422	0.67
**MYO7A**	rs3758708	11	76572111	intron	T	0.109	0.90	0.112	0.92	0.108	0.85
**MYO7A**	rs7105374	11	76541520	intron	A	0.410	0.37	0.426	0.93	0.403	0.32
**MYO7A**	rs7123925	11	76598363	intron	G	0.450	0.61	0.442	0.74	0.454	0.74
**MYO7A**	rs762667	11	76546020	coding exon	C	0.329	0.55	0.327	0.72	0.331	0.21
**MYO7A**	rs883223	11	76602522	intron	A	0.331	0.58	0.333	0.63	0.327	0.84
**MYO7A**	rs885442	11	76597686	non-coding exon	T	0.434	0.51	0.444	0.68	0.425	0.53
**MYO7A**	rs948962	11	76597126	coding exon	A	0.472	0.90	0.467	0.65	0.476	0.63
**MYO7A**	rs948970	11	76563049	intron	G	0.464	0.87	0.465	0.66	0.465	0.44
**NF1**	rs1013948	17	26554835	intron	G	0.144	0.93	0.131	0.84	0.149	0.96
**NF1**	rs10438801	17	26717911	intron	G	0.362	0.77	0.351	0.36	0.369	0.68
**NF1**	rs2953014	17	26520021	intron	G	0.218	0.35	0.219	0.70	0.217	0.17
**NF1**	rs2953016	17	26518819	intron	G	0.196	0.25	0.200	0.24	0.192	0.59
**NRAS**	rs14804	1	115051366	3' UTR	A	0.229	0.40	0.224	0.82	0.237	0.46
**NRAS**	rs8453	1	115061122	5' upstream	T	0.113	0.99	0.115	0.99	0.111	0.90
**NRAS**	rs926938	1	115041339	5' upstream	G	0.476	0.70	0.468	0.19	0.477	0.36
**NRAS**	rs969273	1	115058192	intron	G	0.344	0.25	0.351	0.98	0.346	0.09
**PAX3**	rs12620338	2	222773012	3' UTR	A	0.203	0.50	0.212	0.95	0.196	0.56
**PAX3**	rs12623857	2	222870133	coding exon	A	0.145	0.41	0.160	0.43	0.131	0.82
**PAX3**	rs13405641	2	222869850	intron	A	0.261	0.27	0.286	0.07	0.239	0.99
**PAX3**	rs16863657	2	222872762	5' upstream	G	0.127	0.93	0.108	0.86	0.140	0.73
**PAX3**	rs2033806	2	222777323	intron	T	0.087	0.53	0.075	0.90	0.094	0.70
**PAX3**	rs2276630	2	222866283	intron	A	0.103	0.93	0.087	0.53	0.118	0.89
**PAX3**	rs3770210	2	222871006	intron	A	0.088	0.92	0.080	0.62	0.096	0.74
**PAX3**	rs7559271	2	222776530	intron	G	0.394	0.52	0.390	0.22	0.398	0.71
**PCNA**	rs17349	20	5047516	intron	A	0.130	0.82	0.130	0.94	0.130	0.87
**PCNA**	rs3729558	20	5043321	3' downstream	G	0.434	0.81	0.426	0.97	0.438	0.90
**PLDN**	rs12909221	15	43690071	3' downstream	C	0.440	0.68	0.458	0.82	0.422	0.50
**PLDN**	rs16945097	15	43667414	intron	T	0.134	0.70	0.118	0.89	0.146	0.60
**POMC**	rs1866146	2	25234077	3' downstream	G	0.331	**0.04**	0.336	0.17	0.326	0.09
**POMC**	rs6734859	2	25233412	3' UTR	T	0.148	0.87	0.149	0.69	0.146	0.74
**POMC**	rs7565877	2	25239568	intron	G	0.112	0.69	0.111	0.40	0.113	0.94
**POMC**	rs934778	2	25242728	intron	G	0.356	0.08	0.351	0.72	0.358	**0.03**
**PRKAR1A**	rs11651687	17	64029901	intron	A	0.190	0.40	0.190	0.13	0.193	0.96
**PRKAR1A**	rs4968898	17	64017451	5' upstream	G	0.201	0.81	0.204	0.42	0.202	0.70
**PRKAR1A**	rs8066131	17	64016614	intron	G	0.295	0.66	0.292	0.36	0.296	0.92
**PRKAR1A**	rs8076465	17	64024620	intron	A	0.317	0.83	0.305	0.79	0.323	0.71
**PRKAR1A**	rs8905	17	64039397	3' UTR	G	0.151	0.65	0.142	0.69	0.159	0.87
**PTCH1**	rs16909865	9	97247123	3' UTR	G	0.071	0.79	0.077	0.86	0.064	0.90
**PTCH1**	rs2297087	9	97282746	intron	G	0.201	0.10	0.208	0.40	0.199	0.20
**PTCH1**	rs4448343	9	97306191	intron	G	0.281	0.49	0.288	0.92	0.281	0.45
**PTCH1**	rs473902	9	95335790	intron	G	0.095	0.62	0.085	0.67	0.100	0.83
**PTCH1**	rs574688	9	95318745	intron	G	0.300	0.26	0.288	0.74	0.306	0.19
**PTCH2**	rs3795719	1	45061114	intron	G	0.137	0.44	0.137	0.57	0.137	0.13
**PTCH2**	rs7554177	1	45077697	intron	A	0.309	0.40	0.315	0.52	0.301	0.37
**RAB27A**	rs1007912	15	53360818	intron	A	0.082	0.54	0.071	0.64	0.089	0.61
**RAB27A**	rs1061824	15	53283255	3' UTR	C	0.184	1.00	0.177	0.80	0.192	0.72
**RAB27A**	rs11071175	15	53347717	intron	G	0.482	0.19	0.479	0.14	0.482	0.88
**RAB27A**	rs11855084	15	53368975	intron	A	0.067	0.86	0.076	0.87	0.061	0.95

**RAB27A**	rs12050885	15	53345916	intron	G	0.170	0.94	0.196	0.47	0.147	0.47
**RAB27A**	rs16976177	15	53296552	intron	G	0.155	**0.004**	0.150	**0.01**	0.161	**0.01**
**RAB27A**	rs16976194	15	53307051	intron	T	0.435	0.89	0.454	0.71	0.423	0.75
**RAB27A**	rs17238192	15	53302238	intron	T	0.108	0.21	0.115	0.49	0.106	0.27
**RAB27A**	rs7167572	15	53362172	intron	T	0.359	**0.05**	0.363	0.10	0.359	0.33
**RAB27A**	rs7496857	15	53342428	intron	T	0.253	0.74	0.273	0.56	0.237	0.91
**RAB27A**	rs9920165	15	53291631	intron	G	0.113	0.86	0.101	0.87	0.122	0.68
**RAB38**	rs1027027	11	87486711	3' UTR	A	0.240	0.59	0.252	0.97	0.231	0.61
**RAB38**	rs11602163	11	87548841	5' upstream	G	0.323	0.78	0.326	0.77	0.315	0.70
**RAB38**	rs12295107	11	87548384	5' upstream	A	0.077	0.80	0.081	0.79	0.074	0.94
**RAB38**	rs12576251	11	87521837	intron	G	0.335	0.68	0.336	0.78	0.328	0.57
**RAB38**	rs302646	11	87548096	coding exon	A	0.110	0.85	0.103	0.66	0.115	0.83
**RAB38**	rs524121	11	87487215	intron	C	0.053	0.55	0.051	0.57	0.054	0.74
**RAB38**	rs9144	11	87486405	3' UTR	T	0.408	0.74	0.422	0.85	0.392	0.61
**RAB38**	rs9666730	11	87547390	intron	T	0.159	0.75	0.166	0.76	0.155	0.75
**RABGGTA**	rs3940231	14	23818203	5' upstream	T	0.403	0.75	0.395	0.26	0.406	0.30
**RABGGTA**	rs941505	14	23803112	3' downstream	T	0.080	0.40	0.071	0.72	0.088	0.42
**RGS1**	rs10921202	1	190813675	intron	T	0.054	0.72	0.045	0.72	0.058	0.79
**RGS1**	rs1359062	1	190808095	5' upstream	G	0.181	0.60	0.188	0.99	0.177	0.35
**RGS1**	rs1923949	1	190814333	intron	G	0.273	0.93	0.276	0.19	0.273	0.35
**RGS1**	rs2816306	1	190814668	intron	C	0.111	0.50	0.112	0.53	0.113	0.60
**RGS20**	rs10958392	8	54917377	intron	T	0.429	0.91	0.425	0.49	0.437	0.36
**RGS20**	rs1123133	8	54942667	intron	G	0.034	0.76	0.031	0.86	0.035	0.81
**RGS20**	rs11783652	8	55021047	intron	A	0.359	0.51	0.353	0.18	0.359	0.78
**RGS20**	rs11783925	8	54933413	intron	T	0.239	0.57	0.246	0.74	0.232	0.59
**RGS20**	rs2220093	8	54963880	intron	C	0.104	0.89	0.114	0.83	0.100	0.99
**RGS20**	rs4738519	8	55031026	intron	C	0.138	0.76	0.140	0.38	0.137	0.81
**RGS20**	rs6473895	8	54925457	5' upstream	A	0.063	0.66	0.062	0.83	0.067	0.68
**RGS20**	rs6981243	8	55029044	intron	C	0.423	0.81	0.444	0.95	0.404	0.83
**RGS20**	rs7824575	8	54984872	intron	A	0.238	0.47	0.238	0.54	0.240	0.69
**SILV**	rs10783775	12	54625812	intron	G	0.195	0.86	0.185	0.63	0.198	0.68
**SILV**	rs2069398	12	54647143	5' upstream	A	0.079	0.83	0.067	0.99	0.089	0.77
**SILV**	rs2291615	12	54621417	intron	A	0.248	0.51	0.265	0.99	0.240	0.36
**SLC45A2**	rs35401	5	33977113	3' downstream	G	0.365	0.17	0.376	0.28	0.357	0.65
**SLC45A2**	rs35403	5	33979349	3' downstream	C	0.473	0.97	0.496	0.64	0.459	0.81
**SLC45A2**	rs35405	5	33981515	intron	T	0.455	0.32	0.457	0.24	0.450	0.68
**SLC45A2**	rs35414	5	34005385	intron	T	0.409	0.15	0.417	0.31	0.408	0.36
**SLC45A2**	rs35415	5	34008288	non-coding exon	A	0.429	**0.04**	0.438	0.54	0.427	0.05
**SLC45A2**	rs3756464	5	34005451	intron	A	0.438	0.46	0.428	0.94	0.446	0.29
**SLC45A2**	rs7718382	5	34004647	intron	A	0.197	0.83	0.196	0.62	0.199	0.64
**SNAI2**	rs1992375	8	50000397	5' upstream	A	0.492	0.62	0.483	0.98	0.477	0.48
**SNAI2**	rs2735455	8	49992695	3' downstream	T	0.075	0.72	0.074	0.69	0.075	0.93
**SNX10**	rs13222190	7	26102101	5' upstream	A	0.338	0.41	0.320	0.37	0.353	0.69
**SNX10**	rs1406754	7	26362723	intron	T	0.369	0.05	0.362	0.30	0.377	0.05
**SNX10**	rs1468286	7	26385234	3' downstream	T	0.429	0.35	0.436	0.79	0.429	0.17
**SNX10**	rs2699808	7	26175747	intron	C	0.470	0.65	0.487	0.58	0.455	0.29
**SNX10**	rs3801890	7	26159394	intron	C	0.349	0.99	0.343	0.89	0.347	0.94
**SNX10**	rs7782538	7	26174753	intron	T	0.192	0.77	0.189	0.90	0.200	0.61
**SOX10**	rs139879	7	36689052	3' downstream	T	0.386	0.49	0.394	0.41	0.381	0.99
**SOX11**	rs17362772	2	5748369	5' upstream	G	0.106	0.84	0.091	0.84	0.113	0.74
**SOX11**	rs3922853	2	5748050	5' upstream	A	0.175	0.85	0.155	0.69	0.191	0.97
**SOX11**	rs4371338	2	5788628	3' UTR	A	0.387	0.74	0.369	0.70	0.401	0.59
**SOX11**	rs4669779	2	5795648	3' downstream	C	0.422	0.78	0.406	0.84	0.435	0.99
**SOX11**	rs6432221	2	5746153	5' upstream	T	0.392	0.45	0.405	0.47	0.373	0.52
**TYR**	rs1042602	11	88551344	coding exon	A	0.459	0.75	0.452	0.34	0.470	0.26
**TYR**	rs11018535	11	88591995	intron	C	0.050	0.89	0.041	0.79	0.058	1.00
**TYR**	rs12270717	11	88551838	intron	C	0.259	0.77	0.272	0.14	0.245	0.62
**TYR**	rs17174064	11	88619139	intron	C	0.069	0.56	0.068	0.76	0.071	0.63

**TYR**	rs2186640	11	88615811	intron	G	0.377	0.57	0.392	0.18	0.362	0.11
**TYR**	rs5021654	11	88550237	5' upstream	C	0.373	0.48	0.386	0.21	0.361	0.10
**TYRP1**	rs10809828	9	12697861	intron	G	0.275	0.64	0.281	0.70	0.264	0.82
**TYRP1**	rs11791497	9	12677872	5' upstream	C	0.056	0.48	0.061	0.71	0.052	0.70
**TYRP1**	rs17346161	9	12695162	intron	T	0.053	0.83	0.047	1.00	0.060	0.76
**TYRP1**	rs683	9	12699305	3' UTR	C	0.411	0.70	0.424	0.69	0.396	0.99
**WNT1/WNT10B**	rs3782353	12	47645147	3' downstream	A	0.427	0.81	0.421	0.46	0.431	0.53
**WNT1/WNT10B**	rs7311091	12	47669474	3' downstream	T	0.079	0.47	0.071	0.85	0.084	0.39
**WNT1/WNT10B**	rs833820	12	47634086	3' downstream	C	0.404	0.46	0.402	0.36	0.405	0.99
**WNT1/WNT10B**	rs833839	12	47653157	5' upstream	T	0.468	0.31	0.464	0.18	0.470	0.90
**WNT3A**	rs697763	1	226259245	5' upstream	C	0.332	0.83	0.321	0.08	0.340	0.33
**WNT3A**	rs708122	1	226283620	intron	A	0.356	0.55	0.365	0.45	0.350	0.17
**WNT3A**	rs766972	1	226311447	intron	G	0.298	0.33	0.284	0.07	0.310	0.80
**WNT3A**	rs947631	1	226290170	intron	C	0.398	0.42	0.378	**0.04**	0.413	0.74

Chr, Chromosome; mA, Minor Allele; MAF, minor allele frequency; HWE, Hardy-Weinberg equilibrium

Bold indicates statistically significant results. * *P* -value significant after Bonferroni correction. *** SNPs located in chromosome X

Table S2. List of SNPs associated with pigmentation traits in females and males

				Eye Colour				Skin Colour				Hair Colour			
				Female		Male		Female		Male		Female		Male	
Gene	SNP #rs	Chr	mA	p-value	OR	p-value	OR	p-value	OR	p-value	OR	p-value	OR	p-value	OR
ADAM17	rs12473402	2	C	0.22	1.26 (0.86-1.85)	0.27	1.26 (0.83-1.90)	0.45	1.30 (0.66-2.56)	0.76	1.09 (0.63-1.86)	0.54	0.92 (0.71-1.19)	**0.027**	0.72 (0.54-0.97)
	rs12992105	2	C	0.39	0.86 (0.61-1.22)	**0.022**	1.54 (1.07-2.24)	0.81	0.89 (0.36-2.20)	0.47	1.17 (0.77-1.77)	0.63	1.11 (0.73-1.70)	0.56	1.18 (0.68-2.05)
	rs4258773	2	G	0.45	1.17 (0.78-1.76)	**0.048**	1.34 (1.00-1.80)	0.62	0.94 (0.73-1.20)	0.14	0.72 (0.47-1.11)	0.13	1.54 (0.89-2.68)	0.25	1.43 (0.79-2.60)
ADAMTS20	rs1510521	12	C	**0.025**	1.36 (1.04-1.78)	0.24	1.27 (0.85-1.90)	0.18	0.70 (0.42-1.17)	0.19	1.29 (0.88-1.90)	0.19	1.26 (0.89-1.78)	0.081	1.60 (0.94-2.72)
	rs275630	12	A	**0.012**	1.42 (1.08-1.87)	0.73	0.88 (0.44-1.77)	0.15	0.66 (0.37-1.16)	0.53	0.81 (0.42-1.57)	0.35	1.18 (0.83-1.68)	0.21	1.28 (0.87-1.88)
	rs7297057	12	T	**0.030**	0.67 (0.46-0.97)	0.69	0.81 (0.27-2.37)	0.26	0.49 (0.14-1.75)	0.42	0.87 (0.62-1.22)	0.051	0.61 (0.37-1.02)	0.37	0.81 (0.50-1.30)
AP3B1	rs10514134	5	A	0.46	0.83 (0.51-1.36)	0.25	1.32 (0.82-2.12)	0.43	0.49 (0.08-2.96)	0.54	0.48 (0.04-5.34)	0.36	0.76 (0.41-1.41)	**0.048**	1.86 (1.03-3.38)
	rs11742673	5	A	0.16	0.75 (0.51-1.11)	0.077	1.61 (0.95-2.73)	0.54	0.93 (0.72-1.18)	0.45	1.23 (0.73-2.07)	0.23	1.42 (0.81-2.48)	**0.022**	1.56 (1.06-2.30)
	rs11746090	5	A	0.41	0.86 (0.59-1.24)	**0.023**	1.47 (1.05-2.05)	0.15	0.60 (0.30-1.20)	0.30	1.61 (0.65-3.97)	0.19	1.28 (0.88-1.85)	0.1	1.42 (0.94-2.15)
	rs12657894	5	A	0.12	1.23 (0.95-1.59)	**0.027**	0.63 (0.42-0.95)	0.75	1.06 (0.75-1.51)	0.62	1.10 (0.74-1.65)	0.41	1.15 (0.82-1.61)	0.27	0.74 (0.44-1.26)
	rs13172957	5	G	0.068	1.41 (0.97-2.06)	**0.012**	0.60 (0.40-0.89)	0.37	0.78 (0.46-1.33)	0.55	0.89 (0.60-1.31)	0.08	1.54 (0.94-2.52)	0.38	0.79 (0.47-1.33)
	rs17191796	5	G	0.60	0.88 (0.56-1.40)	0.15	1.38 (0.89-2.16)	0.43	0.49 (0.08-2.96)	0.29	0.32 (0.03-3.11)	0.65	0.88 (0.50-1.54)	**0.028**	1.93 (1.09-3.42)
	rs2636986	5	A	0.61	0.91 (0.62-1.33)	0.29	0.58 (0.21-1.63)	0.41	0.73 (0.34-1.55)	0.59	0.78 (0.32-1.92)	**0.021**	0.60 (0.38-0.95)	0.15	0.71 (0.44-1.14)
	rs389110	5	A	0.61	0.91 (0.62-1.33)	0.29	0.58 (0.21-1.63)	0.52	0.78 (0.37-1.65)	0.59	0.78 (0.32-1.92)	**0.035**	0.63 (0.40-0.99)	0.15	0.71 (0.44-1.14)
	rs6453374	5	A	0.27	0.81 (0.56-1.17)	0.072	1.34 (0.97-1.84)	0.72	0.94 (0.67-1.32)	0.89	0.97 (0.66-1.43)	**0.017**	2.34 (1.20-4.57)	0.3	1.24 (0.83-1.87)
AP3D1	rs10413398	19	C	0.17	0.77 (0.30-1.98)	**0.032**	1.45 (1.03-2.03)	**0.038**	0.40 (0.17-0.97)	0.55	1.31 (0.54-3.17)	0.08	0.24 (0.03-1.83)	0.34	1.23 (0.81-1.89)
	rs17604954	19	A	0.29	0.78 (0.50-1.24)	0.2600	1.27 (0.84-1.90)	**0.019**	0.63 (0.43-0.93)	0.41	1.18 (0.80-1.75)	0.45	0.81 (0.46-1.42)	0.96	0.98 (0.53-1.84)
	rs7256735	19	G	0.46	0.84 (0.52-1.34)	0.32	1.31 (0.77-2.22)	**0.0058**	0.53 (0.34-0.83)	0.5	1.18 (0.73-1.90)	0.69	0.88 (0.46-1.67)	0.81	0.92 (0.48-1.79)
ASIP	rs819133	20	T	0.26	0.78 (0.50-1.21)	0.058	0.66 (0.43-1.01)	**0.046**	0.70 (0.49-0.99)	0.23	0.79 (0.53-1.17)	0.11	0.65 (0.37-1.13)	**0.012**	0.46 (0.23-0.89)
BCL2	rs1462129	18	T	0.17	0.84 (0.65-1.08)	0.52	1.10 (0.82-1.47)	0.42	1.17 (0.80-1.70)	0.30	1.27 (0.80-2.03)	**0.014**	0.45 (0.22-0.89)	0.50	1.24 (0.67-2.26)
	rs1564483	18	A	0.11	1.27 (0.95-1.69)	0.30	1.24 (0.83-1.85)	0.11	1.26 (0.95-1.67)	0.13	0.49 (0.19-1.26)	**0.021**	2.55 (1.21-5.39)	0.66	1.12 (0.66-1.88)
	rs949037	18	T	0.2	0.85 (0.65-1.09)	0.16	1.43 (0.88-2.34)	0.18	1.28 (0.89-1.85)	0.075	1.56 (0.95-2.54)	**0.0083**	0.39 (0.18-0.84)	0.21	1.49 (0.81-2.74)
BCL2A1	rs11636338	15	C	**0.0021**	0.56 (0.39-0.81)	0.87	0.95 (0.52-1.75)	0.38	0.89 (0.68-1.16)	0.25	0.71 (0.39-1.28)	0.81	0.94 (0.58-1.52)	0.17	1.67 (0.83-3.38)
	rs17215263	15	G	**0.033**	0.71 (0.52-0.98)	0.89	0.98 (0.72-1.33)	**0.0023**	0.36 (0.18-0.70)	0.37	0.75 (0.39-1.42)	0.85	1.04 (0.71-1.52)	0.37	0.79 (0.46-1.33)
BLOC1S3	rs7253652	19	C	0.61	0.87 (0.52-1.46)	0.22	1.31 (0.85-2.03)	0.24	1.30 (0.84-2.02)	**0.034**	1.60 (1.03-2.48)	0.54	0.81 (0.41-1.60)	**0.020**	2.04 (1.14-3.66)
CDH1	rs1801552	16	T	0.72	0.95 (0.71-1.27)	0.39	0.83 (0.54-1.27)	0.16	0.76 (0.51-1.11)	0.44	1.13 (0.82-1.56)	0.69	0.90 (0.53-1.52)	**0.012**	1.71 (1.13-2.58)
CDK2	rs2069398	12	A	**0.023**	0.56 (0.32-0.97)	0.6	0.86 (0.48-1.53)	0.42	0.83 (0.53-1.30)	0.79	1.08 (0.62-1.86)	0.72	0.93 (0.51-1.67)	0.19	0.57 (0.24-1.38)
CDKN2A	rs2518719	9	G	0.11	1.33 (0.94-1.87)	0.65	0.92 (0.63-1.33)	0.81	1.04 (0.74-1.45)	**0.0006**	1.87 (1.29-2.69)	0.63	1.12 (0.72-1.74)	0.52	1.17 (0.74-1.84)
	rs2811712	9	G	0.077	0.22 (0.03-1.71)	0.54	0.88 (0.57-1.35)	0.35	1.23 (0.80-1.89)	**0.0044**	0.52 (0.33-0.82)	0.26	0.73 (0.41-1.29)		
	rs3218020	9	T	0.27	1.29 (0.82-2.03)	0.1	1.27 (0.95-1.69)	0.7	1.05 (0.82-1.35)	0.14	1.36 (0.91-2.05)	**0.029**	1.46 (1.04-2.06)	0.059	1.75 (0.96-3.20)
CDKN2B	rs495490	9	C	0.29	1.24 (0.83-1.84)	0.088	0.67 (0.42-1.07)	0.11	1.44 (0.91-2.26)	**0.017**	1.75 (1.10-2.79)	0.65	1.15 (0.64-2.06)	0.21	0.67 (0.35-1.28)
CNO	rs4689527	4	G	0.50	1.22 (0.69-2.14)	**0.048**	1.34 (1.00-1.80)	0.80	1.06 (0.65-1.74)	0.38	1.18 (0.81-1.73)	0.32	1.32 (0.76-2.30)	0.56	1.12 (0.76-1.66)
CTNNBIP1	rs11828	1	G	0.31	0.60 (0.22-1.66)	0.54	0.89 (0.63-1.28)	0.11	0.75 (0.52-1.07)	0.079	0.43 (0.16-1.14)	0.77	0.83 (0.24-2.87)	**0.036**	0.53 (0.29-0.98)
	rs12128766	1	C	0.14	1.35 (0.90-2.02)	**0.0049**	0.43 (0.23-0.80)	**0.047**	0.64 (0.42-0.99)	**0.0001**	0.34 (0.19-0.60)	0.4	0.81 (0.49-1.32)	**0.035**	0.66 (0.44-0.98)
	rs2379107	1	G	0.38	1.19 (0.81-1.75)	0.3	0.82 (0.55-1.21)	0.41	0.63 (0.21-1.90)	0.24	0.52 (0.17-1.58)	0.49	0.84 (0.50-1.40)	**0.024**	0.52 (0.28-0.96)
	rs4846104	1	G	**0.029**	0.54 (0.30-0.97)	0.62	0.86 (0.48-1.56)	0.68	0.90 (0.54-1.50)	0.78	0.92 (0.53-1.62)	0.67	0.85 (0.40-1.79)	0.17	0.54 (0.21-1.39)
EDNRB	rs3818416	13	T	0.15	0.57 (0.25-1.27)	0.14	1.35 (0.90-2.01)	0.78	0.91 (0.47-1.77)	0.28	1.18 (0.88-1.58)	0.16	0.46 (0.14-1.53)	**0.004**	0.12 (0.02-0.92)
F2RL1	rs2243010	5	T	0.17	1.32 (0.89-1.97)	**0.035**	0.64 (0.42-0.98)	0.43	0.55 (0.12-2.47)	0.23	0.38 (0.07-1.99)	0.44	1.22 (0.73-2.04)	0.64	0.88 (0.52-1.50)
GNA11	rs10407783	19	T	0.12	0.59 (0.29-1.19)	**0.017**	2.08 (1.14-3.80)	0.49	1.13 (0.80-1.60)	0.67	0.94 (0.70-1.25)	0.26	1.32 (0.81-2.15)	0.16	1.48 (0.85-2.56)
	rs2238625	19	C	0.92	1.01 (0.78-1.32)	0.28	1.27 (0.82-1.97)	0.39	1.22 (0.78-1.91)	0.62	0.94 (0.72-1.22)	0.094	1.59 (0.91-2.79)	**0.031**	1.94 (1.03-3.63)
	rs308039	19	T	0.55	0.90 (0.65-1.26)	**0.016**	0.58 (0.37-0.91)	0.88	0.98 (0.72-1.33)	0.16	1.29 (0.90-1.86)	**0.040**	0.57 (0.32-0.99)	0.3	0.77 (0.46-1.29)
	rs3786947	19	T	0.24	0.72 (0.41-1.27)	0.062	1.32 (0.99-1.78)	0.91	0.98 (0.69-1.40)	0.81	0.97 (0.65-1.45)	0.06	1.40 (0.99-1.98)	**0.0021**	2.52 (1.35-4.70)
	rs404632	19	T	0.24	0.77 (0.49-1.20)	**0.027**	0.60 (0.37-0.96)	0.39	1.17 (0.82-1.66)	0.16	1.40 (0.87-2.25	0.094	0.60 (0.32-1.12)	0.28	0.72 (0.38-1.34)
GNAQ	rs10781468	9	T	0.57	1.21 (0.64-2.28)	0.43	1.18 (0.79-1.75)	0.77	1.04 (0.80-1.36)	**0.0018**	0.29 (0.13-0.67)	0.088	1.36 (0.96-1.93)	0.26	1.34 (0.80-2.26)
	rs17786974	9	T	0.71	0.91 (0.55-1.51)	0.71	0.91 (0.55-1.51)	0.92	0.98 (0.61-1.56)	0.29	1.24 (0.83-1.85)	**0.014**	0.38 (0.16-0.90)	0.17	0.61 (0.28-1.30)
	rs2296937	9	G	0.15	1.33 (0.90-1.97)	**0.038**	1.64 (1.03-2.61)	0.66	0.73 (0.18-2.96)	**0.0048**	0.32 (0.14-0.74)	0.89	0.96 (0.57-1.63)	0.13	1.56 (0.90-2.71)
	rs3780302	9	T	0.49	0.85 (0.54-1.34)	0.87	0.97 (0.63-1.48)	0.35	1.20 (0.82-1.77)	**0.021**	0.57 (0.35-0.92)	0.72	1.10 (0.66-1.83)	0.65	1.14 (0.66-1.95)
	rs3858119	9	G	0.91	1.04 (0.53-2.01)	0.28	0.65 (0.30-1.45)	0.38	0.86 (0.61-1.21)	**0.0007**	0.27 (0.12-0.61)	0.28	1.30 (0.81-2.09)	0.67	1.12 (0.66-1.89)
	rs4745672	9	T	0.22	0.85 (0.65-1.10)	0.084	0.64 (0.39-1.07)	0.23	1.27 (0.86-1.87)	**0.0029**	0.31 (0.13-0.70)	0.47	0.82 (0.49-1.39)	0.49	0.81 (0.45-1.45)
GNAS	rs12625436	20	A	0.48	1.10 (0.85-1.42)	0.67	1.13 (0.65-1.96)	**0.022**	1.33 (1.04-1.70)	0.65	0.94 (0.70-1.25)	0.064	1.64 (0.96-2.82)	0.18	0.59 (0.26-1.34)
	rs13831	20	T	0.35	0.84 (0.58-1.21)	0.29	0.84 (0.61-1.16)	0.24	1.47 (0.77-2.81)	**0.015**	2.69 (1.16-6.23)	0.73	0.87 (0.36-2.13)	0.3	1.24 (0.83-1.85)
	rs234623	20	G	0.17	1.21 (0.92-1.59)	0.74	1.05 (0.79-1.39)	0.5	1.09 (0.85-1.41)	**0.012**	0.56 (0.35-0.88)	0.39	1.27 (0.72-2.24)	0.54	0.82 (0.43-1.55)
	rs6026561	20	C	0.53	0.84 (0.49-1.45)	0.42	1.26 (0.72-2.19)	**0.0031**	0.58 (0.41-0.84)	0.95	0.99 (0.66-1.48)	**0.0010**	0.45 (0.28-0.73)	0.32	0.82 (0.55-1.21)
	rs6092704	20	C	0.36	0.79 (0.48-1.31)	0.057	0.58 (0.32-1.03)	**0.024**	0.62 (0.41-0.94)	0.64	1.13 (0.68-1.86)	0.14	0.62 (0.32-1.21)	0.21	0.62 (0.29-1.36)
	rs6123832	20	T	0.49	0.84 (0.52-1.37)	**0.038**	0.63 (0.41-0.97)	**0.016**	0.64 (0.44-0.92)	0.71	1.08 (0.71-1.64)	**0.023**	0.67 (0.47-0.95)	0.35	0.77 (0.45-1.33)

GPR143	rs2521667	X	G	0.54	1.13 (0.76-1.68)	0.86	0.95 (0.54-1.67)	0.17	0.55 (0.24-1.29)	0.062	1.66 (0.97-2.86)	0.1	0.68 (0.43-1.10)	**0.0014**	2.80 (1.53-5.13)
	rs2732872	X	C	**0.022**	0.29 (0.09-0.98)	0.80	0.94 (0.55-1.58)	0.26	0.84 (0.63-1.13)	0.5	1.09 (0.85-1.40)	0.26	0.47 (0.11-2.02)	**0.004**	1.57 (1.17-2.11)
HPS1	rs1061135	10	T	0.65	0.91 (0.61-1.36)	0.38	1.23 (0.78-1.95)	**0.015**	0.62 (0.42-0.91)	0.38	0.82 (0.52-1.28)	0.11	0.66 (0.40-1.09)	0.1	0.74 (0.51-1.07)
	rs1739	10	C	0.12	0.61 (0.32-1.17)	0.65	1.15 (0.62-2.12)	0.13	0.66 (0.38-1.14)	0.18	0.66 (0.37-1.21)	0.49	0.75 (0.33-1.73)	**0.0046**	0.19 (0.05-0.82)
	rs1886728	10	C	0.08	1.49 (0.96-2.32)	0.13	0.66 (0.38-1.15)	0.36	0.82 (0.53-1.25)	**0.027**	0.56 (0.34-0.94)	0.54	1.18 (0.69-2.02)	0.094	0.63 (0.37-1.08)
	rs7921146	10	A	0.3	0.60 (0.22-1.65)	0.85	1.03 (0.74-1.44)	**0.017**	0.64 (0.45-0.92)	0.69	1.07 (0.78-1.46)	0.17	0.70 (0.42-1.18)	0.27	0.78 (0.50-1.23)
HPS4	rs17401652	22	T	**0.0001**	0.36 (0.20-0.63)	0.31	1.27 (0.80-2.02)	0.72	0.92 (0.60-1.41)	0.85	0.95 (0.58-1.56)	0.47	0.79 (0.42-1.50)	0.35	0.71 (0.35-1.47)
	rs3747129	22	A	0.33	1.20 (0.83-1.73)	0.8	1.06 (0.69-1.61)	0.31	0.83 (0.57-1.20)	**0.040**	1.58 (1.02-2.46)	0.74	1.09 (0.66-1.81)	0.23	1.42 (0.81-2.49)
	rs9608491	22	G	**0.014**	1.53 (1.09-2.14)	0.23	0.55 (0.20-1.53)	0.26	0.54 (0.19-1.59)	**0.033**	0.65 (0.44-0.97)	0.72	1.10 (0.67-1.80)	0.84	0.88 (0.25-3.07)
HPS5	rs12218	11	G	0.64	0.91 (0.61-1.36)	0.46	1.19 (0.75-1.88)	**0.0061**	0.55 (0.36-0.85)	0.19	0.75 (0.48-1.16)	0.33	1.19 (0.84-1.67)	0.11	1.61 (0.91-2.83)
	rs2049129	11	G	0.84	1.04 (0.70-1.55)	0.11	1.36 (0.93-1.98)	0.14	1.32 (0.91-1.93)	0.43	1.16 (0.80-1.68)	0.64	0.90 (0.56-1.43)	**0.013**	1.80 (1.14-2.83)
	rs2305564	11	A	0.88	0.98 (0.75-1.27)	0.12	1.44 (0.91-2.26)	0.24	0.86 (0.67-1.10)	0.35	0.82 (0.54-1.25)	0.33	1.31 (0.75-2.28)	**0.030**	1.88 (1.08-3.27)
	rs7131332	11	G	0.51	0.88 (0.61-1.28)	0.19	1.49 (0.83-2.68)	**0.0069**	1.61 (1.14-2.28)	0.52	0.88 (0.60-1.29)	0.14	0.52 (0.20-1.34)	**0.033**	2.16 (1.10-4.24)
HPS6	rs3737243	10	A	0.22	0.74 (0.46-1.20)	0.40	0.80 (0.47-1.35)	0.55	1.13 (0.75-1.70)	0.47	1.19 (0.74-1.94)	**0.047**	0.51 (0.26-0.99)	0.64	1.17 (0.61-2.24)
	rs3816	10	G	0.091	2.28 (0.89-5.84)	**0.030**	0.63 (0.42-0.96)	0.19	1.96 (0.69-5.59)	0.19	0.58 (0.26-1.32)	0.28	0.77 (0.47-1.26)	0.65	0.91 (0.59-1.40)
	rs6584475	10	C	0.19	1.42 (0.85-2.39)	**0.020**	1.63 (1.08-2.48)	0.24	1.36 (0.81-2.28)	0.23	1.19 (0.90-1.58)	0.45	1.30 (0.67-2.49)	0.2	1.28 (0.88-1.87)
HRK	rs884378	12	T	0.49	0.77 (0.37-1.63)	0.39	0.87 (0.63-1.20)	0.55	1.09 (0.83-1.43)	0.41	1.36 (0.65-2.86)	**0.032**	0.64 (0.42-0.98)	0.19	0.75 (0.48-1.16)
KIT	rs1008658	4	A	0.24	0.80 (0.55-1.16)	0.89	1.04 (0.58-1.88)	0.24	1.37 (0.80-2.33)	**0.0210**	2.01 (1.10-3.68)	0.44	0.74 (0.34-1.62)	0.63	1.20 (0.57-2.53)
	rs13135792	4	C	0.74	1.09 (0.65-1.83)	0.57	1.12 (0.75-1.68)	0.66	1.06 (0.82-1.35)	0.38	1.29 (0.73-2.29)	0.075	1.76 (0.97-3.21)	**0.013**	1.60 (1.11-2.32)
	rs2237025	4	A	0.16	0.75 (0.51-1.12)	0.33	0.78 (0.47-1.29)	0.35	1.20 (0.82-1.74)	0.43	0.89 (0.68-1.18)	0.36	0.75 (0.40-1.42)	**0.0057**	0.46 (0.27-0.79)
	rs2298976	4	C	0.088	0.68 (0.43-1.07)	0.62	0.89 (0.57-1.40)	0.96	1.01 (0.68-1.50)	**0.020**	1.68 (1.08-2.61)	0.059	0.55 (0.29-1.06)	0.72	1.10 (0.66-1.82)
	rs4864920	4	T	**0.047**	0.68 (0.46-0.99)	0.31	1.55 (0.68-3.54)	0.38	1.51 (0.60-3.79)	0.29	1.19 (0.86-1.65)	0.06	0.65 (0.41-1.04)	0.53	1.40 (0.50-3.89)
	rs6554198	4	G	0.79	0.95 (0.64-1.40)	0.46	1.17 (0.77-1.79)	0.18	1.28 (0.89-1.84)	0.077	0.65 (0.40-1.05)	**0.026**	0.43 (0.19-0.97)	**0.008**	0.35 (0.15-0.84)
	rs759083	4	G	0.45	1.11 (0.85-1.45)	0.36	0.75 (0.40-1.40)	0.52	0.85 (0.52-1.39)	0.63	1.10 (0.74-1.63)	**0.016**	1.52 (1.08-2.14)	0.054	1.45 (0.99-2.13)
KITLG	rs10858753	12	T	0.62	1.16 (0.65-2.06)	0.092	1.70 (0.92-3.15)	**0.022**	0.53 (0.30-0.91)	0.15	1.58 (0.84-2.96)	0.32	0.66 (0.28-1.57)	0.097	1.92 (0.92-4.04)
LYST	rs6429238	1	T	**0.024**	0.65 (0.45-0.95)	0.21	0.68 (0.37-1.26)	0.62	1.14 (0.67-1.96)	0.33	0.75 (0.43-1.33)	0.49	0.85 (0.53-1.36)	0.66	1.09 (0.75-1.58)
	rs6699717	1	A	**0.041**	0.65 (0.43-0.98)	0.26	1.31 (0.81-2.12)	0.44	1.16 (0.79-1.72)	0.85	0.96 (0.61-1.51)	0.76	1.09 (0.63-1.88)	0.22	1.27 (0.87-1.85)
	rs7541057	1	C	**0.043**	0.66 (0.44-0.98)	0.37	0.81 (0.50-1.30)	0.35	1.20 (0.82-1.76)	0.78	0.96 (0.73-1.27)	0.51	1.16 (0.67-2.00)	0.15	0.76 (0.52-1.11)
MCAM	rs2249466	11	T	0.42	1.19 (0.79-1.79)	**0.0014**	1.67 (1.22-2.31)	**0.019**	0.45 (0.23-0.88)	0.18	1.32 (0.88-2.00)	0.17	1.45 (0.85-2.47)	0.45	1.23 (0.71-2.13)
	rs2511837	11	T	0.32	1.14 (0.88-1.47)	**0.020**	0.71 (0.53-0.95)	0.94	1.02 (0.68-1.52)	**0.018**	0.71 (0.54-0.94)	0.087	1.64 (0.91-2.93)	0.68	0.92 (0.64-1.35)
	rs6589732	11	A	0.59	0.87 (0.53-1.43)	0.11	0.79 (0.58-1.06)	0.41	1.11 (0.87-1.42)	**0.019**	0.71 (0.53-0.95)	0.052	0.47 (0.22-1.02)	0.53	1.25 (0.63-2.49)
MCOLN3	rs10782537	1	C	0.73	0.86 (0.35-2.09)	0.30	0.61 (0.24-1.59)	0.21	1.70 (0.73-3.99)	**0.025**	0.37 (0.15-0.92)	0.51	1.42 (0.52-3.91)	0.25	0.77 (0.49-1.22)
	rs12030837	1	T	**0.045**	0.63 (0.41-0.98)	0.69	0.72 (0.14-3.74)	0.063	5.24 (0.64-42.93)	0.27	0.80 (0.53-1.19)	0.57	0.84 (0.47-1.52)	0.84	0.95 (0.55-1.64)
	rs12735211	1	A	0.28	1.32 (0.80-2.19)	0.46	1.21 (0.73-2.01)	0.23	1.35 (0.82-2.23)	0.31	1.30 (0.78-2.15)	0.67	1.15 (0.60-2.21)	**0.029**	1.93 (1.10-3.41)
	rs2304641	1	A	0.76	0.94 (0.63-1.41)	0.56	0.71 (0.22-2.30)	**0.0027**	9.93 (1.29-76.46)	0.38	0.86 (0.60-1.21)	0.45	1.69 (0.46-6.29)	0.087	0.61 (0.34-1.09)
	rs6674050	1	A	0.087	1.91 (0.92-3.98)	**0.036**	1.42 (1.02-1.97)	0.38	0.85 (0.60-1.21)	0.054	1.37 (0.99-1.90)	0.11	2.08 (0.89-4.82)	**0.019**	1.63 (1.09-2.44)
MITF	rs11128152	3	T	**0.016**	0.61 (0.41-0.92)	0.87	1.03 (0.71-1.49)	0.54	1.37 (0.50-3.75)	0.58	0.89 (0.60-1.33)	0.32	0.77 (0.46-1.30)	0.49	0.84 (0.51-1.38)
MLANA	rs1056796	9	T	0.23	0.68 (0.35-1.30)	0.73	0.88 (0.44-1.78)	0.58	1.08 (0.83-1.40)	**0.026**	1.55 (1.05-2.28)	**0.043**	1.44 (1.01-2.05)	0.76	1.08 (0.64-1.83)
	rs10758717	9	C	0.79	1.04 (0.77-1.42)	0.74	1.05 (0.77-1.45)	0.19	1.26 (0.89-1.78)	**0.030**	1.54 (1.04-2.27)	0.41	1.50 (0.59-3.81)	0.48	1.21 (0.72-2.03)
	rs2150702	9	C	0.66	0.91 (0.61-1.37)	0.32	1.15 (0.87-1.51)	0.67	1.10 (0.72-1.68)	**0.012**	1.71 (1.12-2.62)	0.16	0.78 (0.55-1.10)	0.058	1.79 (0.96-3.36)
	rs7872509	9	C	0.17	0.76 (0.51-1.13)	0.11	1.32 (0.94-1.87)	0.28	1.22 (0.85-1.76)	0.15	1.29 (0.92-1.81)	0.87	0.96 (0.58-1.59)	**0.035**	1.77 (1.05-3.00)
MLPH	rs10173589	2	G	0.66	0.90 (0.60-1.36)	0.52	0.86 (0.55-1.35)	0.37	0.86 (0.61-1.20)	0.18	1.28 (0.89-1.84)	**0.016**	0.49 (0.27-0.91)	0.43	1.21 (0.76-1.92)
	rs13383648	2	C	0.33	1.24 (0.81-1.88)	0.88	1.04 (0.64-1.68)	0.41	1.19 (0.79-1.78)	**0.029**	0.62 (0.40-0.96)	0.73	0.92 (0.55-1.52)	0.97	1.01 (0.57-1.80)
MUTED	rs2743989	6	T	0.68	0.95 (0.72-1.24)	0.14	1.37 (0.90-2.08)	0.28	0.77 (0.47-1.24)	0.086	1.41 (0.95-2.10)	**0.023**	0.41 (0.17-0.98)	0.31	1.21 (0.84-1.74)
	rs2748376	6	T	0.41	1.19 (0.74-1.93)	0.17	0.75 (0.49-1.13)	**0.032**	0.61 (0.38-0.96)	**0.038**	1.34 (1.01-1.78)	0.11	1.31 (0.94-1.84)	0.16	1.61 (0.85-3.06)
	rs2815155	6	C	0.45	1.20 (0.75-1.93)	0.55	0.92 (0.69-1.22)	0.21	1.26 (0.88-1.83)	**0.003**	0.46 (0.27-0.77)	0.31	0.71 (0.36-1.40)	**0.034**	0.66 (0.45-0.98)
MYO7A	rs762667	11	C	0.43	0.86 (0.60-1.25)	0.13	1.64 (0.87-3.10)	**0.035**	0.57 (0.34-0.96)	0.18	1.54 (0.81-2.93)	0.44	0.74 (0.34-1.62)	0.055	1.46 (0.99-2.15)
	rs948970	11	G	0.13	0.74 (0.50-1.09)	0.25	0.75 (0.46-1.24)	0.57	0.89 (0.59-1.34)	0.86	0.98 (0.74-1.28)	**0.010**	0.43 (0.22-0.87)	0.11	0.57 (0.28-1.16)
NF1	rs1013948	17	G	**0.032**	1.47 (1.04-2.08)	0.25	1.27 (0.85-1.91)	0.34	1.21 (0.82-1.78)	0.14	1.39 (0.89-2.18)	0.41	1.21 (0.77-1.88)	0.055	0.53 (0.27-1.05)
	rs10438801	17	G	0.51	1.09 (0.84-1.43)	**0.032**	1.56 (1.04-2.35)	0.84	0.97 (0.76-1.25)	**0.0220**	1.38 (1.05-1.83)	0.88	1.03 (0.73-1.45)	0.89	0.95 (0.44-2.04)
	rs2953016	17	G	0.078	1.33 (0.97-1.82)	0.54	1.31 (0.57-3.02)	0.33	1.16 (0.86-1.58)	0.069	1.35 (0.97-1.87)	0.28	1.25 (0.84-1.85)	**0.011**	0.47 (0.25-0.87)
NRAS	rs14804	1	T	0.054	2.05 (1.00-4.21)	0.71	1.06 (0.76-1.48)	0.083	1.96 (0.89-4.32)	0.093	1.32 (0.95-1.82)	**0.0013**	3.77 (1.76-8.05)	0.12	1.39 (0.92-2.11)
PAX3	rs12623857	2	A	**0.021**	1.55 (1.07-2.23)	0.30	0.36 (0.04-3.09)	0.39	0.85 (0.60-1.22)	**0.0007**	0.51 (0.34-0.76)	0.52	1.17 (0.73-1.88)	0.88	0.96 (0.54-1.69)
	rs16863657	2	G	0.2	0.77 (0.52-1.15)	0.46	1.20 (0.74-1.95)	0.68	0.92 (0.62-1.36)	**0.019**	0.60 (0.38-0.92)	0.079	0.62 (0.35-1.09)	0.067	1.75 (0.98-3.15)
PLDN	rs12909221	15	C	0.36	0.88 (0.67-1.16)	**0.0026**	2.00 (1.26-3.18)	0.63	0.89 (0.55-1.44)	**0.018**	0.56 (0.35-0.91)	0.11	1.66 (0.91-3.02)	0.17	1.50 (0.82-2.75)
POMC	rs934778	2	C	0.48	1.15 (0.79-1.67)	0.12	1.39 (0.92-2.10)	0.17	1.28 (0.90-1.82)	**0.005**	0.66 (0.49-0.88)	0.31	1.29 (0.79-2.11)	0.43	1.24 (0.73-2.12)
PRKAR1A	rs8066131	17	G	**0.033**	1.93 (1.06-3.50)	0.48	0.75 (0.33-1.68)	0.30	1.39 (0.75-2.57)	0.24	1.21 (0.88-1.65)	0.073	1.96 (0.97-3.93)	0.25	1.36 (0.81-2.30)
PTCH2	rs3795719	1	G	0.12	0.69 (0.44-1.10)	0.25	0.78 (0.51-1.20)	0.88	1.03 (0.68-1.56)	**0.010**	0.56 (0.36-0.88)	0.41	1.26 (0.73-2.17)	0.88	0.96 (0.55-1.66)

RAB27A	rs11071175	15	G	0.17	0.75 (0.50-1.13)	0.16	0.70 (0.42-1.16)	**0.0058**	0.71 (0.56-0.91)	0.58	0.92 (0.70-1.22)	0.30	0.76 (0.46-1.27)	0.12	0.58 (0.29-1.19)
	rs17238192	15	T	0.37	0.81 (0.50-1.30)	0.69	1.09 (0.72-1.66)	**0.0095**	0.60 (0.41-0.89)	0.46	1.16 (0.78-1.75)	0.44	0.78 (0.41-1.48)	0.26	1.35 (0.81-2.24)
	rs7167572	15	T	0.13	1.54 (0.89-2.64)	0.073	0.55 (0.28-1.08)	**0.015**	1.39 (1.06-1.81)	0.18	0.66 (0.36-1.21)	0.22	1.54 (0.79-2.98)	0.41	1.38 (0.65-2.92)
	rs7496857	15	T	0.66	0.92 (0.63-1.34)	0.23	0.63 (0.28-1.38)	**0.011**	0.38 (0.18-0.82)	0.42	0.85 (0.58-1.25)	0.84	0.95 (0.59-1.54)	0.83	0.96 (0.63-1.44)
RAB38	rs1027027	11	A	0.3	1.17 (0.87-1.58)	0.41	1.39 (0.64-3.02)	**0.012**	2.78 (1.18-6.56)	0.27	1.55 (0.71-3.38)	**0.018**	2.76 (1.25-6.07)	0.29	1.32 (0.79-2.22)
	rs9666730	11	T	0.16	1.28 (0.91-1.80)	0.58	1.36 (0.46-3.99)	0.78	0.95 (0.64-1.39)	0.4	1.17 (0.82-1.67)	**0.026**	3.54 (1.25-10.02)	0.33	1.26 (0.80-2.00)
RABGGTA	rs3940231	14	A	0.29	0.81 (0.56-1.19)	0.14	0.81 (0.61-1.08)	0.73	0.96 (0.75-1.23)	**0.0059**	0.57 (0.38-0.85)	0.12	1.51 (0.89-2.56)	0.55	0.85 (0.50-1.44)
RGS1	rs1359062	1	G	0.54	0.73 (0.26-2.04)	0.74	0.94 (0.65-1.35)	**0.011**	0.30 (0.11-0.80)	0.47	0.86 (0.57-1.29)	**0.025**	3.19 (1.23-8.27)	0.4	0.81 (0.50-1.33)
RGS20	rs10958392	8	T	0.10	1.40 (0.93-2.11)	0.10	1.51 (0.92-2.49)	0.89	1.03 (0.71-1.49)	0.19	1.20 (0.91-1.57)	0.065	1.66 (0.95-2.91)	**0.049**	1.86 (1.02-3.37)
	rs11783925	8	T	0.34	1.50 (0.66-3.41)	**0.022**	0.67 (0.47-0.95)	0.47	0.88 (0.62-1.25)	0.14	0.50 (0.20-1.28)	0.64	0.75 (0.22-2.57)	0.091	0.63 (0.37-1.09)
	rs6981243	8	C	0.054	0.77 (0.58-1.01)	0.081	1.55 (0.95-2.52)	**0.020**	0.57 (0.35-0.91)	0.66	0.90 (0.56-1.45)	0.32	1.29 (0.78-2.15)	0.16	0.76 (0.52-1.12)
	rs7824575	8	A	0.18	1.29 (0.89-1.86)	0.58	0.79 (0.33-1.86)	0.29	1.17 (0.88-1.56)	0.051	0.43 (0.18-1.03)	0.056	1.59 (0.99-2.55)	**0.015**	1.92 (1.13-3.25)
SLC45A2	rs35414	5	T	0.30	0.82 (0.56-1.20)	0.075	0.76 (0.57-1.03)	0.20	0.79 (0.54-1.13)	**0.015**	0.70 (0.53-0.94)	0.27	0.82 (0.58-1.17)	**0.0078**	0.49 (0.29-0.82)
	rs35415	5	A	0.19	1.38 (0.86-2.22)	0.21	0.83 (0.62-1.11)	0.51	0.92 (0.71-1.19)	**0.022**	0.72 (0.54-0.95)	0.41	0.76 (0.38-1.49)	**0.024**	0.54 (0.32-0.91)
SNAI2	rs1992375	8	A	0.86	0.96 (0.64-1.45)	0.056	0.76 (0.57-1.01)	0.20	1.28 (0.88-1.88)	**0.0048**	0.68 (0.51-0.89)	0.34	0.75 (0.41-1.37)	**0.048**	0.57 (0.33-0.99)
	rs2735455	8	A	0.91	0.97 (0.60-1.58)	0.1	1.56 (0.92-2.65)	0.059	1.57 (0.97-2.54)	**0.0054**	2.15 (1.23-3.75)	0.90	1.04 (0.55-1.99)	0.085	1.80 (0.94-3.45)
SNX10	rs1406754	7	T	0.087	0.60 (0.33-1.10)	0.15	1.35 (0.89-2.04)	**0.024**	0.55 (0.33-0.93)	**0.044**	1.50 (1.01-2.22)	0.76	0.95 (0.66-1.36)	0.53	1.19 (0.69-2.03)
	rs2699808	7	C	0.17	1.38 (0.87-2.17)	0.33	0.80 (0.52-1.25)	**0.022**	1.68 (1.06-2.67)	0.65	0.94 (0.72-1.22)	0.81	1.08 (0.59-1.95)	0.14	0.65 (0.38-1.13)
SOX11	rs17362772	2	G	0.16	0.72 (0.46-1.15)	0.064	1.61 (0.98-2.65)	0.56	0.88 (0.58-1.34)	0.75	1.08 (0.68-1.72)	**0.031**	0.52 (0.27-0.99)	0.22	1.45 (0.81-2.59)
	rs6432221	2	T	**0.042**	0.68 (0.47-0.98)	0.28	1.17 (0.88-1.57)	0.27	0.82 (0.57-1.17)	0.86	1.04 (0.69-1.55)	0.22	0.80 (0.56-1.15)	0.65	1.09 (0.75-1.60)
SOX4	rs9368326	6	G	**0.011**	0.21 (0.05-0.92)	0.41	0.59 (0.16-2.21)	0.31	1.20 (0.84-1.73)	**0.0017**	5.00 (1.08-23.10)	0.33	1.22 (0.82-1.83)		
TYR	rs1042602	11	A	0.30	0.81 (0.54-1.21)	**0.0062**	0.47 (0.26-0.83)	0.61	1.03 (0.81-1.32)	0.056	0.62 (0.38-1.02)	0.095	1.58 (0.93-2.68)	0.52	0.80 (0.40-1.60)
	rs12270717	11	C	0.37	1.15 (0.85-1.54)	**0.037**	1.53 (1.02-2.29)	0.43	1.15 (0.81-1.63)	0.1	1.30 (0.95-1.79)	0.26	0.76 (0.47-1.23)	0.24	1.29 (0.85-1.95)
	rs17793678	11	T	0.35	1.15 (0.86-1.55)	**0.025**	1.45 (1.05-2.01)	0.42	1.15 (0.81-1.64)	0.087	1.32 (0.96-1.82)	0.39	0.81 (0.50-1.32)	0.18	1.34 (0.88-2.03)
	rs2186640	11	G	0.66	1.10 (0.66-1.84)	**0.025**	1.62 (1.06-2.49)	0.87	0.99 (0.77-1.27)	0.2	1.20 (0.91-1.60)	0.42	0.82 (0.51-1.32)	0.15	1.51 (0.85-2.67)
	rs5021654	11	C	0.71	1.10 (0.66-1.84)	**0.018**	1.67 (1.09-2.56)	0.94	0.99 (0.78-1.27)	0.12	1.37 (0.92-2.04)	0.49	0.89 (0.63-1.25)	0.19	1.45 (0.83-2.55)
TYRP1	rs10809828	9	G	**0.024**	0.39 (0.16-0.95)	0.56	0.91 (0.67-1.24)	0.61	1.08 (0.82-1.42)	0.13	1.66 (0.83-3.32)	0.53	1.13 (0.78-1.63)	0.40	1.44 (0.63-3.28)
WNT3A	rs708122	1	T	0.11	0.74 (0.51-1.07)	0.55	0.88 (0.59-1.33)	0.3	1.35 (0.77-2.37)	0.85	1.04 (0.70-1.53)	**0.010**	0.27 (0.08-0.89)	0.45	0.82 (0.49-1.38)

Chr, Chromosome; mA, Minor Allele; OR, Odds Ratio per minor allele; CI, Confidence Interval.
Bold indicates statistically significant results

Table S3. List of SNPs associated with sun response traits in females and males

				Sunburns				Lentigines				Naevi			
				Female		Male		Female		Male		Female		Male	
Gene	SNP ID	Chr	mA	p-value	OR	p-value	OR	p-value	OR	p-value	OR	p-value	OR	p-value	OR
ADAM17	rs12473402	2	C	0.74	1.05 (0.80-1.36)	0.75	1.10 (0.60-2.03)	0.26	0.71 (0.40-1.28)	0.14	1.38 (0.90-2.13)	**0.0063**	0.64 (0.46-0.89)	0.23	1.33 (0.83-2.12)
	rs17524425	2	G	**0.022**	1.52 (1.06-2.18)	0.24	0.81 (0.58-1.15)	0.061	1.37 (0.98-1.92)	**0.0004**	0.52 (0.35-0.75)	0.14	1.30 (0.92-1.83)	0.26	0.80 (0.53-1.19)
	rs4258773	2	G	0.96	0.99 (0.77-1.28)	0.40	1.23 (0.76-2.00)	0.25	0.85 (0.64-1.12)	0.094	1.51 (0.93-2.43)	**0.0063**	0.44 (0.24-0.83)	0.11	1.53 (0.91-2.58)
ADAMTS20	rs1510521	12	C	0.43	0.81 (0.48-1.37)	0.36	1.15 (0.86-1.54)	0.52	0.91 (0.68-1.22)	0.077	1.88 (0.92-3.86)	**0.043**	0.72 (0.52-0.99)	0.19	0.74 (0.48-1.16)
	rs2048348	12	A	0.56	0.50 (0.05-5.54)	**0.035**	0.64 (0.41-0.97)	0.26	1.32 (0.81-2.15)	**0.047**	0.63 (0.40-1.00)	0.37	1.28 (0.75-2.17)	0.54	0.85 (0.50-1.45)
	rs2062731	12	G	**0.03**	0.59 (0.36-0.96)	0.82	1.05 (0.67-1.66)	0.69	1.11 (0.66-1.87)	0.23	0.71 (0.40-1.25)	0.16	1.44 (0.87-2.38)	0.69	0.90 (0.53-1.52)
	rs7960952	12	C	0.43	0.84 (0.54-1.30)	0.14	1.23 (0.93-1.63)	0.29	0.80 (0.53-1.21)	0.099	1.60 (0.91-2.84)	**0.036**	0.73 (0.54-0.98)	0.075	0.66 (0.42-1.04)
AP3B1	rs10514134	5	A	0.74	1.07 (0.71-1.63)	**0.0066**	1.98 (1.20-3.28)	0.27	0.40 (0.07-2.19)	0.16	1.46 (0.86-2.48)	0.61	0.87 (0.52-1.47)	0.48	1.22 (0.71-2.11)
	rs10805919	5	C	0.12	0.75 (0.52-1.07)	0.58	0.91 (0.67-1.25)	0.35	0.83 (0.55-1.24)	0.31	0.80 (0.53-1.23)	0.045	0.64 (0.41-0.99)	0.63	0.80 (0.31-2.04)
	rs11742673	5	A	0.79	1.05 (0.72-1.54)	0.41	1.25 (0.74-2.13)	**0.023**	1.74 (1.07-2.83)	0.56	1.19 (0.66-2.12)	**0.023**	1.78 (1.09-2.90)	0.73	0.92 (0.57-1.48)
	rs11746090	5	A	0.16	1.28 (0.90-1.83)	**0.0083**	3.57 (1.28-9.95)	0.47	1.12 (0.82-1.52)	0.11	1.33 (0.93-1.91)	0.17	1.71 (0.81-3.60)	0.42	0.65 (0.21-1.96)
	rs12657894	5	A	**0.016**	1.56 (1.09-2.23)	0.35	1.31 (0.74-2.32)	0.26	1.36 (0.79-2.34)	0.29	0.71 (0.38-1.33)	0.09	1.63 (0.94-2.82)	0.63	1.12 (0.70-1.78)
	rs13172957	5	G	0.057	1.41 (0.99-2.01)	0.4	1.32 (0.69-2.55)	0.42	0.78 (0.43-1.42)	0.83	0.97 (0.70-1.33)	**0.019**	1.44 (1.06-1.96)	0.59	1.13 (0.72-1.77)
	rs2636986	5	A	**0.01**	0.62 (0.43-0.89)	**0.024**	3.08 (1.09-8.71)	0.055	0.72 (0.52-1.01)	0.13	2.18 (0.76-6.26)	**0.0054**	0.58 (0.39-0.87)	0.61	0.90 (0.62-1.33)
	rs34436	5	G	0.089	0.69 (0.45-1.06)	**0.014**	0.54 (0.33-0.89)	0.53	1.17 (0.71-1.93)	0.058	0.63 (0.39-1.02)	0.79	1.07 (0.63-1.82)	0.56	1.15 (0.71-1.86)
	rs389110	5	A	**0.01**	0.62 (0.43-0.89)	**0.024**	3.08 (1.09-8.71)	**0.036**	0.38 (0.15-0.97)	0.13	2.18 (0.76-6.26)	**0.005**	0.58 (0.39-0.87)	0.61	0.90 (0.62-1.33)
	rs4703747	5	G	0.051	0.72 (0.52-1.00)	0.32	1.20 (0.84-1.72)	0.15	0.74 (0.49-1.11)	0.41	1.65 (0.49-5.60)	**0.014**	0.56 (0.35-0.90)	0.63	1.10 (0.74-1.64)
	rs6453373	5	A	**0.047**	0.61 (0.37-1.00)	**0.015**	0.52 (0.31-0.90)	0.41	1.28 (0.71-2.34)	0.17	0.67 (0.38-1.19)	0.86	0.95 (0.52-1.73)	0.12	1.56 (0.91-2.67)
	rs6453374	5	A	0.35	1.18 (0.83-1.68)	**0.022**	2.49 (1.10-5.61)	0.068	1.83 (0.94-3.57)	0.26	1.21 (0.86-1.71)	**0.019**	2.20 (1.18-4.09)	0.49	0.88 (0.61-1.27)
AP3D1	rs2240655	19	T	0.93	1.02 (0.68-1.52)	0.24	1.31 (0.84-2.06)	0.34	0.81 (0.53-1.24)	0.072	0.64 (0.40-1.04)	0.89	1.03 (0.65-1.65)	**0.031**	0.56 (0.32-0.96)
	rs3786971	19	TT	0.67	0.92 (0.63-1.35)	0.25	0.78 (0.51-1.19)	0.44	0.90 (0.68-1.18)	0.088	0.67 (0.42-1.06)	**0.032**	1.75 (1.06-2.87)	0.80	1.08 (0.61-1.90)
AP3M2	rs7009632	8	G	0.54	1.16 (0.73-1.85)	0.64	1.14 (0.67-1.94)	0.41	0.84 (0.56-1.27)	0.35	1.31 (0.74-2.29)	0.52	1.10 (0.82-1.50)	**0.0045**	2.29 (1.31-4.02)
	rs7823824	8	A	0.47	1.18 (0.75-1.87)	0.77	1.05 (0.78-1.40)	0.18	0.75 (0.50-1.14)	0.68	1.12 (0.65-1.93)	0.75	1.05 (0.78-1.41)	**0.0021**	1.91 (1.12-3.28)
ASIP	rs6142129	20	G	0.57	0.85 (0.48-1.50)	0.052	0.53 (0.27-1.02)	0.26	1.25 (0.84-1.86)	**0.0041**	0.53 (0.35-0.82)	0.64	0.90 (0.59-1.38)	0.4	1.16 (0.83-1.61)
BCL2	rs1462129	18	T	0.39	1.19 (0.80-1.75)	0.76	1.08 (0.67-1.72)	0.067	0.64 (0.39-1.03)	**0.0075**	1.93 (1.19-3.14)	0.19	0.70 (0.41-1.20)	0.06	0.58 (0.33-1.04)
	rs949037	18	T	0.61	0.89 (0.57-1.38)	0.73	1.09 (0.67-1.78)	0.16	0.70 (0.42-1.16)	**0.013**	1.78 (1.13-2.81)	0.069	0.60 (0.33-1.06)	0.11	0.77 (0.56-1.06)
CDH1	rs11075699	16	G	0.19	0.74 (0.48-1.16)	0.37	1.30 (0.75-2.27)	0.69	0.94 (0.71-1.25)	0.47	1.25 (0.68-2.27)	0.50	0.86 (0.55-1.34)	**0.031**	1.43 (1.03-1.98)
	rs2276329	16	G	0.87	1.05 (0.58-1.90)	**0.039**	1.96 (1.02-3.75)	0.48	1.29 (0.64-2.59)	0.13	1.59 (0.86-2.93)	0.20	0.59 (0.26-1.37)	0.58	0.56 (0.06-4.85)
CDH3	rs1124770	16	A	0.82	0.96 (0.67-1.38)	0.69	1.08 (0.74-1.57)	**0.038**	0.65 (0.44-0.98)	0.56	1.36 (0.32-5.77)	0.57	1.14 (0.72-1.82)	0.15	0.28 (0.03-2.18)
	rs1886700	16	A	0.25	1.22 (0.86-1.73)	0.096	0.21 (0.02-1.77)	**0.032**	1.52 (1.03-2.26)	0.72	1.08 (0.70-1.69)	0.74	0.93 (0.61-1.42)	0.57	0.56 (0.06-4.83)
CDK2	rs2069398	12	A	**0.028**	0.61 (0.39-0.95)	0.5	0.83 (0.47-1.44)	**0.031**	0.59 (0.36-0.96)			0.27	0.73 (0.40-1.31)	0.64	0.86 (0.45-1.65)
CDK4	rs2069502	12	A	0.090	0.73 (0.51-1.05)	**0.013**	1.52 (1.09-2.13)	0.063	0.74 (0.53-1.02)	0.37	0.82 (0.54-1.26)	0.57	0.88 (0.57-1.36)	0.64	0.86 (0.45-1.65)
	rs2270777	12	G	0.12	0.73 (0.50-1.09)	**0.037**	1.61 (1.03-2.53)	**0.037**	0.74 (0.56-0.98)	0.59	0.88 (0.55-1.40)	0.15	0.80 (0.59-1.08)	0.70	1.11 (0.67-1.84)
CDKN2A	rs3218020	9	T	**0.012**	1.65 (1.12-2.43)	0.16	1.44 (0.86-2.40)	0.12	0.68 (0.42-1.10)	0.11	0.64 (0.37-1.10)	0.81	0.94 (0.55-1.60)	0.88	0.96 (0.53-1.72)
	rs3731239	9	C	0.58	0.85 (0.47-1.52)	0.28	0.68 (0.33-1.37)	**0.021**	1.58 (1.07-2.34)	0.60	1.22 (0.58-2.55)	0.85	1.09 (0.55-2.15)	0.61	1.22 (0.58-2.55)
CDKN2B	rs495490	9	C	0.31	0.50 (0.12-2.01)	**0.049**	0.64 (0.41-1.00)	0.57	1.62 (0.29-8.94)	0.24	0.75 (0.47-1.21)	0.54	0.84 (0.49-1.46)	0.9	0.97 (0.59-1.59)
CLIP1	rs7388	12	A	0.27	1.18 (0.88-1.59)	**0.0063**	1.58 (1.13-2.21)	0.094	1.33 (0.95-1.85)	0.2	1.25 (0.88-1.78)	0.065	1.49 (0.98-2.27)	0.097	1.46 (0.93-2.29)
CTNNBIP1	rs11828	1	G	**0.041**	0.72 (0.52-0.99)	0.31	1.24 (0.82-1.89)	0.27	0.79 (0.53-1.20)	0.087	0.42 (0.15-1.16)	0.3	0.79 (0.50-1.24)	0.31	0.78 (0.48-1.26)
	rs12128766	1	C	0.2	0.85 (0.66-1.09)	0.75	1.05 (0.79-1.39)	0.86	0.96 (0.58-1.57)	0.086	0.60 (0.34-1.08)	**0.033**	0.62 (0.40-0.96)	0.37	1.24 (0.77-1.99)
	rs2379107	1	G	0.068	1.42 (0.97-2.07)	0.51	0.86 (0.55-1.34)	**0.0021**	0.09 (0.01-0.68)	0.13	1.37 (0.91-2.07)	0.17	2.28 (0.73-7.12)	0.25	1.28 (0.84-1.94)
EDNRB	rs11149080	13	G	0.81	0.94 (0.59-1.51)	0.58	1.08 (0.82-1.43)	**0.020**	0.56 (0.34-0.91)	0.43	1.20 (0.76-1.88)	0.69	1.06 (0.79-1.43)	0.1	1.31 (0.95-1.81)
	rs2242991	5	G	0.67	1.20 (0.53-2.73)	0.51	1.15 (0.76-1.76)	0.20	1.31 (0.86-2.00)	**0.038**	1.63 (1.02-2.59)	0.17	0.73 (0.46-1.15)	0.06	1.45 (0.99-2.12)
	rs2243010	5	T	0.088	0.20 (0.02-1.71)	**0.034**	0.65 (0.43-0.97)	0.39	0.84 (0.57-1.25)	**0.020**	0.60 (0.39-0.93)	0.34	0.80 (0.50-1.28)	0.33	2.16 (0.48-9.82)
	rs2243051	5	G	0.21	0.85 (0.65-1.10)	0.45	0.90 (0.68-1.19)	0.53	1.15 (0.75-1.75)	**0.036**	0.72 (0.53-0.98)	0.84	0.96 (0.60-1.51)	**0.0015**	1.91 (1.14-3.19)
	rs639342	5	A	0.68	1.08 (0.74-1.59)	0.86	0.96 (0.63-1.47)	0.18	0.77 (0.53-1.13)	0.16	1.40 (0.87-2.24)	0.35	1.21 (0.81-1.78)	**0.0058**	0.52 (0.32-0.85)
GNA11	rs308039	19	T	**0.012**	0.61 (0.42-0.90)	0.48	0.88 (0.61-1.27)	0.2	0.80 (0.57-1.13)	0.091	0.68 (0.43-1.07)	0.61	0.91 (0.62-1.33)	0.56	1.13 (0.75-1.70)
	rs404632	19	T	**0.018**	0.60 (0.39-0.92)	0.27	0.77 (0.47-1.23)	0.32	0.53 (0.15-1.89)	0.49	0.54 (0.09-3.26)	0.62	0.88 (0.53-1.47)	0.17	1.39 (0.88-2.19)
GNAQ	rs11145647	9	C	0.43	1.45 (0.54-3.86)	0.28	1.20 (0.86-1.68)	0.19	0.45 (0.13-1.55)	**0.011**	1.79 (1.14-2.80)	0.24	0.79 (0.54-1.17)	**0.026**	1.52 (1.05-2.19)
	rs3858119	9	G	0.19	1.26 (0.89-1.79)	0.45	0.76 (0.37-1.56)	**0.0081**	1.69 (1.14-2.50)	0.82	1.05 (0.69-1.60)	0.22	1.30 (0.85-1.98)	0.54	0.90 (0.63-1.28)
	rs4745672	9	T	**0.009**	0.57 (0.38-0.87)	0.72	1.09 (0.68-1.75)	0.30	0.78 (0.49-1.25)	0.74	0.91 (0.54-1.54)	0.20	0.72 (0.43-1.21)	0.068	1.62 (0.97-2.70)
GNAS	rs234623	20	G	0.50	0.87 (0.58-1.30)	0.072	1.52 (0.96-2.41)	0.37	1.22 (0.79-1.89)	0.17	1.42 (0.86-2.36)	0.53	0.86 (0.54-1.38)	**0.046**	1.70 (1.00-2.92)
	rs6064714	20	G	0.76	1.19 (0.39-3.58)	0.79	0.83 (0.22-3.15)	0.51	0.67 (0.20-2.22)	**0.025**	1.64 (1.05-2.56)	0.92	1.07 (0.29-3.95)	0.74	0.92 (0.55-1.54)
	rs6092704	20	C	**0.0014**	0.46 (0.29-0.75)	0.12	0.66 (0.38-1.12)	0.81	0.94 (0.56-1.57)	**0.019**	0.50 (0.28-0.90)	0.52	1.19 (0.70-2.04)	0.24	0.69 (0.37-1.30)
GPR143	rs2521578	X	A	**0.0077**	1.77 (1.15-2.72)	0.22	0.85 (0.65-1.11)	0.37	0.80 (0.50-1.30)	0.54	1.09 (0.82-1.46)	0.21	0.71 (0.40-1.23)		
	rs2521667	X	G	0.26	0.80 (0.55-1.17)	0.54	1.09 (0.83-1.43)	**0.021**	0.28 (0.09-0.89)	0.80	0.93 (0.52-1.66)	0.082	0.33 (0.08-1.41)	**0.0004**	1.68 (1.27-2.22)
HPS1	rs12242431	10	C	0.28	1.23 (0.84-1.79)	**0.0056**	0.51 (0.31-0.83)	0.21	0.75 (0.48-1.17)	0.11	0.64 (0.38-1.10)	0.82	1.05 (0.66-1.68)	0.67	0.90 (0.53-1.50)
	rs17535384	10	G	0.094	1.40 (0.94-2.09)	**0.020**	1.61 (1.07-2.43)	0.74	1.08 (0.69-1.67)	0.59	0.88 (0.54-1.42)	0.44	0.89 (0.66-1.20)	0.12	1.48 (0.90-2.43)

HPS4	rs1894707	22	C	0.32	0.88 (0.68-1.13)	0.18	1.31 (0.88-1.95)	0.34	1.31 (0.75-2.31)	0.07	1.48 (0.97-2.27)	0.36	1.15 (0.85-1.57)	**0.0094**	2.29 (1.24-4.23)
	rs9608491	22	G	0.28	0.55 (0.18-1.67)	**0.0078**	0.63 (0.45-0.89)	0.11	1.40 (0.92-2.11)	0.17	0.78 (0.55-1.12)	0.68	0.92 (0.62-1.37)	0.18	0.77 (0.52-1.14)
	rs9613187	22	T	0.71	0.75 (0.17-3.39)	**0.025**	0.61 (0.40-0.95)	0.37	1.25 (0.77-2.01)	0.15	0.69 (0.42-1.14)	0.18	0.71 (0.42-1.19)	0.52	0.84 (0.49-1.44)
HPS6	rs3737243	10	A	0.40	1.21 (0.78-1.87)	**0.021**	0.55 (0.33-0.92)	0.95	1.02 (0.62-1.67)	0.55	0.84 (0.48-1.47)	0.17	0.81 (0.60-1.10)	0.62	0.86 (0.48-1.55)
HRK	rs4767462	12	G	0.51	0.84 (0.50-1.41)	0.15	1.48 (0.87-2.53)	**0.020**	0.52 (0.29-0.91)	0.39	0.78 (0.44-1.38)	0.3	0.70 (0.35-1.40)	0.17	1.48 (0.85-2.59)
HTR2B	rs10194776	2	T	**0.031**	0.76 (0.59-0.98)	**0.034**	1.56 (1.03-2.37)	0.40	0.80 (0.48-1.34)	0.39	1.21 (0.78-1.89)	0.84	0.94 (0.54-1.67)	0.14	1.43 (0.89-2.31)
	rs17619600	2	C	0.077	0.65 (0.41-1.05)	0.71	0.91 (0.54-1.53)	0.27	0.74 (0.44-1.26)	0.062	1.68 (0.97-2.94)	0.9	0.97 (0.58-1.61)	**0.018**	1.95 (1.13-3.35)
	rs2161891	2	G	**0.021**	0.73 (0.56-0.96)	0.20	1.50 (0.80-2.80)	0.52	0.91 (0.68-1.21)	0.25	1.29 (0.84-1.97)	0.21	1.47 (0.82-2.66)	0.055	1.56 (0.99-2.48)
	rs4973377	2	A	**0.033**	1.42 (1.03-1.97)	0.14	0.38 (0.10-1.47)	0.28	1.21 (0.85-1.73)	**0.010**	0.17 (0.04-0.80)	0.65	0.92 (0.63-1.34)	0.22	0.76 (0.48-1.19)
KIT	rs1008658	4	A	**0.029**	0.55 (0.32-0.95)	0.56	1.19 (0.67-2.12)	**0.012**	0.69 (0.52-0.92)	0.19	1.50 (0.81-2.80)	0.69	0.94 (0.69-1.28)	0.16	1.57 (0.84-2.92)
	rs13135792	4	C	**0.030**	1.74 (1.05-2.87)	**0.049**	1.49 (1.00-2.21)	0.23	1.39 (0.81-2.38)	0.54	1.14 (0.75-1.75)	0.63	0.86 (0.46-1.61)	0.26	1.20 (0.87-1.65)
	rs4864920	4	T	0.074	0.75 (0.55-1.03)	0.13	0.73 (0.49-1.10)	0.066	0.68 (0.46-1.03)	**0.028**	2.90 (1.05-8.05)	0.23	0.79 (0.53-1.17)	0.39	0.82 (0.52-1.30)
	rs6554198	4	G	**0.011**	0.54 (0.33-0.87)	**0.02**	0.55 (0.33-0.92)	0.064	1.69 (0.96-2.98)	0.12	1.41 (0.91-2.20)	0.49	1.17 (0.74-1.84)	0.49	0.90 (0.66-1.22)
KITLG	rs10858758	12	G	0.64	0.92 (0.64-1.31)	0.066	1.45 (0.98-2.16)	0.64	1.09 (0.77-1.53)	0.35	0.64 (0.24-1.65)	0.35	1.19 (0.83-1.72)	**0.036**	0.61 (0.38-0.98)
LYST	rs11810173	1	T	0.032	0.69 (0.49-0.97)	0.93	1.02 (0.70-1.47)	0.18	1.28 (0.89-1.85)	**0.020**	0.19 (0.04-0.93)	0.23	1.32 (0.84-2.09)	0.052	0.64 (0.41-1.02)
	rs7541057	1	C	0.68	0.92 (0.61-1.38)	0.19	0.83 (0.62-1.10)	0.4	0.82 (0.52-1.30)	**0.017**	1.46 (1.07-2.01)	0.16	0.39 (0.09-1.70)	0.54	1.18 (0.69-2.00)
MCAM	rs2249466	11	T	0.51	1.26 (0.64-2.46)	0.19	1.56 (0.80-3.06)	0.18	1.25 (0.90-1.73)	0.13	1.40 (0.90-2.18)	0.87	0.97 (0.68-1.38)	**0.0039**	2.03 (1.25-3.32)
	rs2511837	11	T	**0.044**	1.53 (1.01-2.31)	0.40	0.83 (0.53-1.29)	0.14	1.40 (0.89-2.22)	0.19	0.71 (0.43-1.18)	0.062	0.65 (0.41-1.02)	0.96	0.99 (0.60-1.63)
	rs6589732	11	A	0.48	1.18 (0.74-1.89)	**0.013**	0.50 (0.28-0.87)	0.4	0.89 (0.68-1.17)	**0.034**	1.61 (1.04-2.51)	0.28	1.27 (0.81-2.00)	0.38	0.75 (0.39-1.44)
MCOLN3	rs10518327	1	A	0.56	1.11 (0.78-1.58)	0.38	0.70 (0.32-1.55)	0.53	1.26 (0.61-2.60)	0.87	0.97 (0.70-1.35)	0.34	1.44 (0.69-2.99)	**0.049**	2.24 (1.02-4.91)
	rs10782537	1	C	0.06	0.75 (0.55-1.01)	0.75	1.14 (0.49-2.66)	0.57	0.89 (0.60-1.33)	0.27	0.78 (0.51-1.21)	0.44	0.66 (0.22-1.97)	**0.045**	0.62 (0.39-1.00)
	rs12030837	1	T	**0.021**	0.63 (0.43-0.94)	0.78	1.06 (0.71-1.59)	0.59	0.88 (0.55-1.41)	0.84	1.05 (0.67-1.63)	0.79	1.07 (0.65-1.76)	0.34	0.77 (0.45-1.33)
	rs7522239	1	A	**0.0012**	0.55 (0.38-0.80)	0.58	1.12 (0.76-1.65)	0.33	0.80 (0.51-1.26)	0.69	1.09 (0.71-1.66)	0.82	0.95 (0.58-1.53)	0.45	0.83 (0.49-1.39)
MITF	rs13072665	3	A	0.41	1.19 (0.79-1.78)	**0.036**	1.85 (1.03-3.32)	0.57	1.14 (0.72-1.79)	**0.0043**	2.49 (1.29-4.78)	0.73	1.10 (0.65-1.85)	0.73	1.11 (0.60-2.04)
MLANA	rs10975339	9	T	0.60	1.15 (0.68-1.94)	0.24	1.27 (0.86-1.87)	0.42	0.85 (0.58-1.26)	0.49	0.89 (0.65-1.23)	**0.0044**	0.33 (0.14-0.78)	0.49	1.17 (0.75-1.83)
	rs2150702	9	C	0.16	0.75 (0.50-1.12)	0.09	1.45 (0.94-2.22)	0.80	0.94 (0.59-1.51)	0.064	0.62 (0.38-1.03)	**0.025**	0.53 (0.29-0.95)	0.064	1.61 (0.96-2.70)
MLPH	rs10173589	2	G	0.67	1.09 (0.74-1.61)	0.33	1.24 (0.80-1.92)	0.13	1.34 (0.91-1.97)	0.38	0.81 (0.51-1.29)	**0.0014**	1.89 (1.29-2.78)	0.43	1.58 (0.52-4.84)
	rs2292881	2	T			**0.011**	2.01 (1.16-3.47)	0.95	0.99 (0.60-1.63)	0.2	0.71 (0.43-1.19)	0.44	1.24 (0.73-2.09)	0.24	1.42 (0.80-2.52)
	rs729389	2	A	0.48	0.60 (0.14-2.53)	0.075	0.69 (0.46-1.04)	0.29	1.29 (0.80-2.06)	**0.017**	0.59 (0.38-0.91)	0.091	1.50 (0.94-2.41)	**0.020**	0.55 (0.32-0.94)
	rs7606177	2	C	0.29	1.55 (0.68-3.51)	0.75	1.13 (0.54-2.34)	0.16	0.52 (0.21-1.30)	**0.023**	0.69 (0.50-0.95)	0.14	1.37 (0.90-2.10)	0.2	0.79 (0.55-1.14)
	rs880931	2	T	0.25	0.40 (0.08-2.06)	0.53	0.86 (0.53-1.39)	0.5	1.18 (0.72-1.93)	**0.035**	0.62 (0.39-0.97)	0.27	1.32 (0.81-2.13)	0.066	0.61 (0.35-1.06)
MUTED	rs2748376	6	T	0.15	0.71 (0.45-1.13)	0.53	0.84 (0.50-1.43)	0.81	0.94 (0.57-1.56)	0.35	1.15 (0.85-1.56)	**0.038**	1.37 (1.02-1.84)	0.27	1.30 (0.81-2.10)
MYO7A	rs11605022	11	G	**0.0077**	1.40 (1.09-1.79)	0.56	1.09 (0.82-1.44)	0.1	1.25 (0.96-1.64)	0.81	0.96 (0.71-1.31)	0.51	1.19 (0.71-2.02)	0.25	0.83 (0.60-1.14)
	rs12793189	11	A	0.21	1.17 (0.91-1.49)	0.075	0.65 (0.40-1.05)	0.52	1.16 (0.74-1.80)	**0.036**	0.59 (0.35-0.97)	**0.021**	1.72 (1.09-2.70)	0.31	0.75 (0.43-1.31)
	rs7105374	11	AA	0.64	1.09 (0.76-1.57)	**0.023**	1.39 (1.04-1.85)	0.56	1.09 (0.83-1.43)	**0.010**	1.47 (1.08-2.01)	0.51	1.19 (0.71-2.02)	0.32	0.89 (0.62-1.28)
	rs7123925	11	G	0.30	0.82 (0.56-1.20)	0.12	0.80 (0.60-1.06)	0.38	0.88 (0.67-1.16)	0.17	0.81 (0.60-1.10)	**0.0049**	0.53 (0.34-0.82)	0.27	0.84 (0.61-1.15)
	rs762667	11	C	**0.016**	1.37 (1.06-1.77)	0.54	0.89 (0.60-1.31)	0.12	1.24 (0.94-1.64)	0.78	0.96 (0.69-1.32)	0.84	0.97 (0.72-1.31)	0.58	0.91 (0.65-1.27)
NF1	rs2953014	17	C	0.42	1.35 (0.64-2.85)	**0.002**	0.18 (0.05-0.64)	0.37	0.86 (0.62-1.19)	0.35	0.84 (0.58-1.21)	0.12	1.40 (0.91-2.13)	**0.038**	1.61 (1.03-2.52)
	rs2953016	17	G	**0.013**	0.30 (0.11-0.84)	0.052	1.39 (0.99-1.95)	0.69	0.92 (0.61-1.38)	0.93	1.02 (0.71-1.45)	0.32	0.56 (0.16-1.91)	0.37	0.81 (0.50-1.30)
NRAS	rs8453	1	A	0.15	0.33 (0.07-1.65)	0.36	1.22 (0.79-1.87)	0.5	0.60 (0.13-2.69	0.68	1.11 (0.66-1.86)	0.13	3.61 (0.72-18.13)	**0.025**	0.52 (0.28-0.95)
PAX3	rs12620338	2	A	0.14	0.76 (0.52-1.10)	0.05	0.67 (0.44-1.00)	**0.017**	0.23 (0.06-0.86)	0.23	0.79 (0.54-1.16)	0.14	0.75 (0.50-1.11)	0.21	1.34 (0.85-2.11)
	rs13405641	2	A	0.07	1.31 (0.98-1.76)	0.64	1.16 (0.61-2.21)	0.013	0.67 (0.48-0.92)	0.15	0.80 (0.58-1.09)	0.35	1.17 (0.84-1.64)	0.83	1.04 (0.74-1.45)
	rs16863657	2	G	**0.0033**	0.57 (0.38-0.83)	0.89	1.03 (0.67-1.59)	0.27	0.79 (0.52-1.20)	0.28	1.30 (0.80-2.11)	**0.0053**	0.50 (0.30-0.84)	0.69	1.11 (0.68-1.81)
	rs7559271	2	G	0.076	1.27 (0.97-1.64)	0.38	0.79 (0.47-1.33)	**0.014**	1.42 (1.07-1.89)	0.22	0.71 (0.41-1.23)	0.082	1.65 (0.95-2.86)	0.86	1.05 (0.59-1.88)
PCNA	rs17349	20	T	0.62	0.90 (0.60-1.36)	0.66	0.90 (0.57-1.42)	0.55	0.88 (0.59-1.33)	0.43	1.20 (0.76-1.88)	**0.029**	0.58 (0.35-0.97)	0.66	1.11 (0.70-1.77)
	rs3729558	20	G	**0.023**	0.75 (0.58-0.96)	0.13	0.67 (0.40-1.13)	0.42	1.23 (0.75-2.02)	0.29	0.74 (0.43-1.29)	0.44	1.20 (0.75-1.89)	0.21	0.68 (0.37-1.26)
PLDN	rs12909221	15	C	0.58	1.15 (0.70-1.89)	**0.022**	1.66 (1.07-2.56)	0.25	1.19 (0.89-1.59)	0.27	1.18 (0.88-1.60)	0.58	1.13 (0.72-1.77)	0.12	1.53 (0.90-2.59)
POMC	rs1866146	2	C	0.42	0.87 (0.61-1.23)	0.077	1.32 (0.97-1.79)	**0.026**	1.42 (1.04-1.95)	0.32	0.70 (0.34-1.42)	0.05	1.39 (1.00-1.92)	0.72	0.87 (0.41-1.85)
	rs6734859	2	T	0.13	0.76 (0.53-1.08)	**0.0095**	1.79 (1.15-2.79)	0.082	1.47 (0.95-2.27)	0.83	1.05 (0.68-1.61)	0.055	1.56 (1.00-2.44)	0.42	0.55 (0.12-2.57)
	rs7565877	2	G	0.37	0.83 (0.55-1.25)	0.78	0.94 (0.59-1.48)	0.38	0.81 (0.50-1.30)	0.65	0.89 (0.55-1.46)	0.078	0.63 (0.37-1.08)	**0.024**	0.51 (0.28-0.94)
PRKAR1A	rs4968898	17	G	0.23	1.22 (0.89-1.67)	0.2	1.25 (0.89-1.77)	**0.022**	1.54 (1.06-2.24)	0.42	1.51 (0.55-4.19)	0.42	0.85 (0.58-1.26)	0.13	0.70 (0.43-1.12)
	rs8066131	17	G	0.14	0.82 (0.63-1.07)	0.20	0.59 (0.27-1.33)	**0.0093**	0.60 (0.40-0.88)	0.69	0.92 (0.60-1.40)	0.77	0.94 (0.62-1.43)	**0.016**	2.67 (1.22-5.81)
PTCH1	rs2297087	9	G	0.32	0.67 (0.31-1.48)	**0.016**	1.66 (1.10-2.50)	0.17	0.80 (0.57-1.11)	0.16	0.52 (0.21-1.30)	**0.032**	1.60 (1.05-2.45)	0.25	1.30 (0.83-2.05)
	rs4448343	9	G	0.48	1.14 (0.79-1.65)	**0.013**	1.70 (1.12-2.59)	0.14	0.79 (0.57-1.08)	0.35	1.18 (0.83-1.69)	**0.026**	1.66 (1.06-2.59)	0.1	1.34 (0.94-1.92)
RAB27A	rs1061824	15	G	**0.048**	1.45 (1.00-2.11)	0.074	1.39 (0.97-2.00)	0.61	1.10 (0.77-1.56)	0.5	1.14 (0.78-1.68)	0.27	1.71 (0.68-4.29)	0.67	0.90 (0.56-1.45)
	rs16976177	15	G	0.089	1.41 (0.95-2.09)	**0.028**	1.48 (1.04-2.12)	0.98	0.99 (0.64-1.54)	0.14	1.45 (0.88-2.38)	0.54	1.12 (0.78-1.62)	0.56	1.32 (0.52-3.34)
	rs16976194	15	T	0.49	0.92 (0.72-1.17)	0.23	1.30 (0.85-2.00)	**0.022**	1.81 (1.08-3.03)	**0.022**	1.72 (1.08-2.76)	0.40	0.83 (0.54-1.28)	0.17	1.42 (0.86-2.36)
RAB38	rs1027027	11	A	0.59	1.08 (0.81-1.44)	0.35	1.45 (0.67-3.14)	0.14	1.27 (0.93-1.74)	0.42	0.87 (0.62-1.22)	**0.019**	1.49 (1.07-2.07)	0.78	0.94 (0.60-1.47)
	rs11602163	11	G	0.19	0.79 (0.56-1.13)	0.20	1.52 (0.80-2.88)	0.37	0.74 (0.38-1.43)	0.078	1.33 (0.97-1.85)	**0.009**	0.57 (0.38-0.87)	0.15	1.65 (0.84-3.25)
	rs12576251	11	G	0.45	1.25 (0.70-2.24)	0.27	1.42 (0.76-2.68)	0.82	0.97 (0.72-1.30)	0.06	1.36 (0.99-1.88)	**0.037**	0.71 (0.51-0.98)	0.09	1.80 (0.93-3.49)
	rs524121	11	C	0.21	0.70 (0.40-1.23)	0.65	1.16 (0.62-2.15)	**0.010**	0.40 (0.20-0.83)	0.31	0.70 (0.35-1.39)	0.32	0.70 (0.34-1.45)	0.36	1.34 (0.72-2.52)

	rs9144	11	C	0.26	1.32 (0.81-2.14)	0.13	1.47 (0.89-2.45)	0.41	0.80 (0.47-1.37)	**0.021**	1.42 (1.05-1.92)	0.072	0.76 (0.56-1.03)	**0.035**	1.80 (1.05-3.07)
RGS1	rs1359062	1	G	0.1	1.37 (0.94-2.00)	0.53	1.12 (0.79-1.60)	**0.017**	3.91 (1.11-13.83)	0.50	0.66 (0.20-2.21)	0.093	1.37 (0.95-1.96)	0.30	0.78 (0.48-1.26)
RGS20	rs1123133	8	G	0.59	0.82 (0.40-1.70)	0.35	1.47 (0.66-3.28)	0.16	1.76 (0.78-3.99)	0.47	0.73 (0.31-1.70)	0.14	1.79 (0.84-3.78)	0.32	1.55 (0.67-3.59)
	rs2220093	8	G	0.41	0.50 (0.09-2.74)	0.13	0.71 (0.45-1.12)	0.56	1.14 (0.74-1.76)	0.22	0.27 (0.03-2.58)	0.42	1.21 (0.76-1.92)	**0.022**	0.51 (0.28-0.93)
	rs6473895	8	A	0.35	1.28 (0.76-2.17)	0.82	0.93 (0.52-1.66)			**0.026**	2.12 (1.07-4.21)	0.37	1.30 (0.73-2.31)	0.26	1.46 (0.76-2.78)
	rs7824575	8	A	0.18	0.60 (0.28-1.29)	**0.029**	2.50 (1.06-5.90)	0.44	0.72 (0.32-1.63)	0.27	1.67 (0.66-4.24)	0.075	0.38 (0.11-1.27)	0.31	0.79 (0.50-1.24)
SLC45A2	rs35405	5	T	0.23	1.17 (0.91-1.50)	0.45	1.18 (0.76-1.84)	0.55	0.86 (0.54-1.39)	**0.018**	1.75 (1.10-2.79)	0.12	0.79 (0.58-1.07)	0.29	1.19 (0.86-1.65)
	rs35414	5	T	0.71	0.93 (0.64-1.35)	**0.02**	0.61 (0.40-0.93)	0.068	1.46 (0.97-2.20)	0.40	0.78 (0.44-1.39)	0.24	1.31 (0.83-2.06)	0.054	0.72 (0.52-1.01)
SNAI2	rs1992375	8	A	0.23	0.86 (0.67-1.10)	0.51	1.14 (0.72-1.81)	0.59	0.89 (0.58-1.37)	0.19	0.82 (0.61-1.10)	**0.0017**	0.41 (0.22-0.75)	0.39	0.80 (0.49-1.32)
	rs2735455	8	A	**0.0096**	1.89 (1.16-3.09)			0.16	1.45 (0.86-2.47)	0.72	0.89 (0.49-1.64)	0.46	1.23 (0.71-2.13)	0.42	0.78 (0.41-1.46)
SNX10	rs1406754	7	T	0.21	1.27 (0.88-1.82)	0.65	1.15 (0.62-2.13)	0.73	1.07 (0.72-1.60)	0.22	0.76 (0.50-1.18)	0.29	1.18 (0.86-1.62)	**0.039**	1.42 (1.02-1.99)
	rs1468286	7	T	0.67	0.91 (0.57-1.43)	**0.03**	1.36 (1.03-1.79)	0.25	1.27 (0.85-1.91)	0.87	0.96 (0.56-1.63)	0.18	0.82 (0.61-1.10)	0.58	1.09 (0.80-1.49)
	rs2699808	7	C	0.19	0.84 (0.65-1.09)	0.49	0.86 (0.55-1.33)	0.52	1.15 (0.75-1.76)	**0.032**	1.73 (1.04-2.89)	0.23	0.76 (0.49-1.18)	0.12	0.78 (0.58-1.07)
SOX11	rs17362772	2	G	**0.034**	0.65 (0.43-0.97)	0.53	1.18 (0.71-1.95)	0.56	0.87 (0.54-1.39)	0.44	1.23 (0.72-2.11)	0.24	0.73 (0.43-1.25)	0.26	0.71 (0.39-1.30)
TYR	rs1042602	11	A	0.62	0.90 (0.59-1.37)	0.20	0.72 (0.44-1.19)	**0.033**	1.68 (1.04-2.71)	**0.0082**	0.66 (0.48-0.90)	0.46	1.20 (0.74-1.95)	**0.031**	0.70 (0.50-0.97)
	rs17174064	11	C	0.7	1.10 (0.67-1.82)	0.21	0.69 (0.39-1.23)	0.28	0.74 (0.44-1.27)	0.86	1.06 (0.57-1.95)	0.47	0.80 (0.43-1.48)	**0.040**	1.87 (1.04-3.34)
	rs2186640	11	G	0.37	0.80 (0.49-1.31)	0.3	1.34 (0.77-2.35)	0.081	0.70 (0.47-1.05)	0.13	1.28 (0.93-1.75)	0.19	1.45 (0.84-2.53)	**0.015**	1.50 (1.08-2.09)
	rs5021654	11	C	0.40	0.81 (0.50-1.32)	0.18	1.47 (0.83-2.60)	0.12	0.73 (0.49-1.08)	0.062	1.35 (0.98-1.87)	0.19	1.46 (0.84-2.55)	**0.019**	1.77 (1.09-2.87)
TYRP1	rs10809828	9	G	0.18	0.82 (0.62-1.10)	0.062	1.94 (0.95-3.95)	0.19	0.81 (0.60-1.10)	**0.0004**	1.82 (1.29-2.57)	0.09	0.43 (0.15-1.25)	0.23	1.31 (0.84-2.05)
	rs11791497	9	C	0.21	0.70 (0.40-1.23)	**0.026**	2.05 (1.08-3.91)	0.41	0.77 (0.40-1.45)			0.54	0.81 (0.41-1.61)	0.78	0.91 (0.48-1.75)
	rs683	9	C	0.1	0.81 (0.63-1.05)	0.17	1.41 (0.86-2.33)	0.40	0.84 (0.56-1.26)	**0.018**	1.97 (1.12-3.47)	0.26	0.84 (0.62-1.14)	**0.027**	1.84 (1.08-3.13)
WNT3A	rs697763	1	C	**0.0086**	1.61 (1.13-2.30)	0.60	0.90 (0.61-1.33)	0.052	1.47 (1.00-2.18)	0.23	1.30 (0.85-1.98)	0.5	0.87 (0.57-1.32)	**0.017**	0.58 (0.37-0.91)
	rs708122	1	T	**0.0003**	0.60 (0.46-0.80)	0.14	1.56 (0.86-2.83)	**0.042**	0.53 (0.29-0.98)	0.49	0.90 (0.67-1.21)	0.80	1.06 (0.69-1.62)	0.15	1.27 (0.91-1.76)
	rs766972	1	G	**0.039**	1.45 (1.02-2.07)	0.59	0.83 (0.43-1.63)	**0.0054**	1.74 (1.17-2.57)	0.12	1.40 (0.92-2.14)	0.32	2.15 (0.51-9.16)	**0.036**	0.62 (0.39-0.97)
	rs947631	1	C	**0.0026**	1.77 (1.22-2.57)	0.16	1.46 (0.86-2.47)	0.13	1.37 (0.91-2.05)	0.072	1.49 (0.96-2.29)	0.35	0.81 (0.52-1.26)	0.15	0.79 (0.58-1.09)

Chr, Chromosome; mA, Minor Allele; OR, Odds Ratio per minor allele; CI, Confidence Interval.

Bold indicates statistically significant results

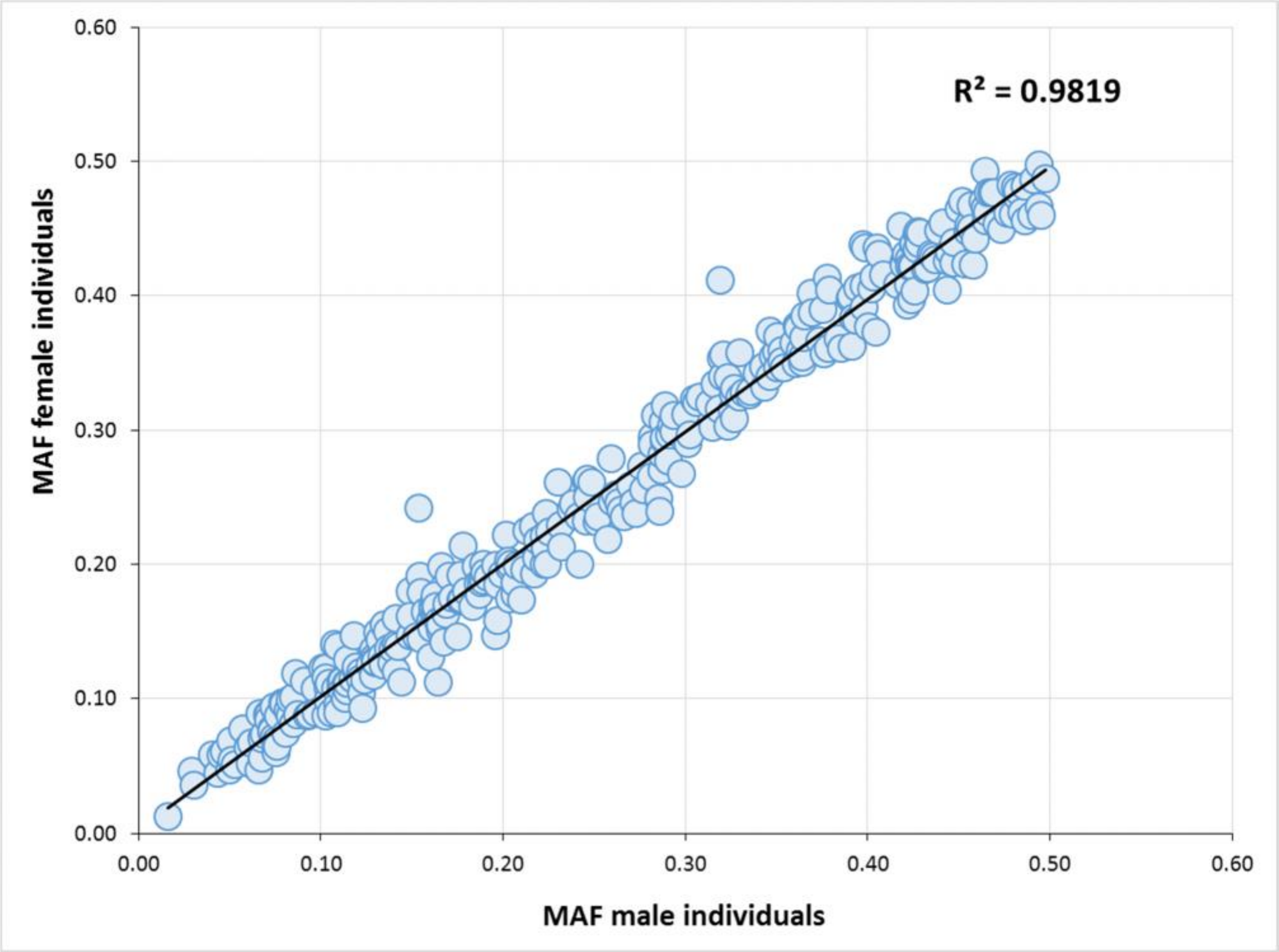

Figure S1. **Comparison of minor allele frequencies, female versus male individuals.** Minor allele frequencies were not significantly different between females and males after Bonferroni correction was applied.

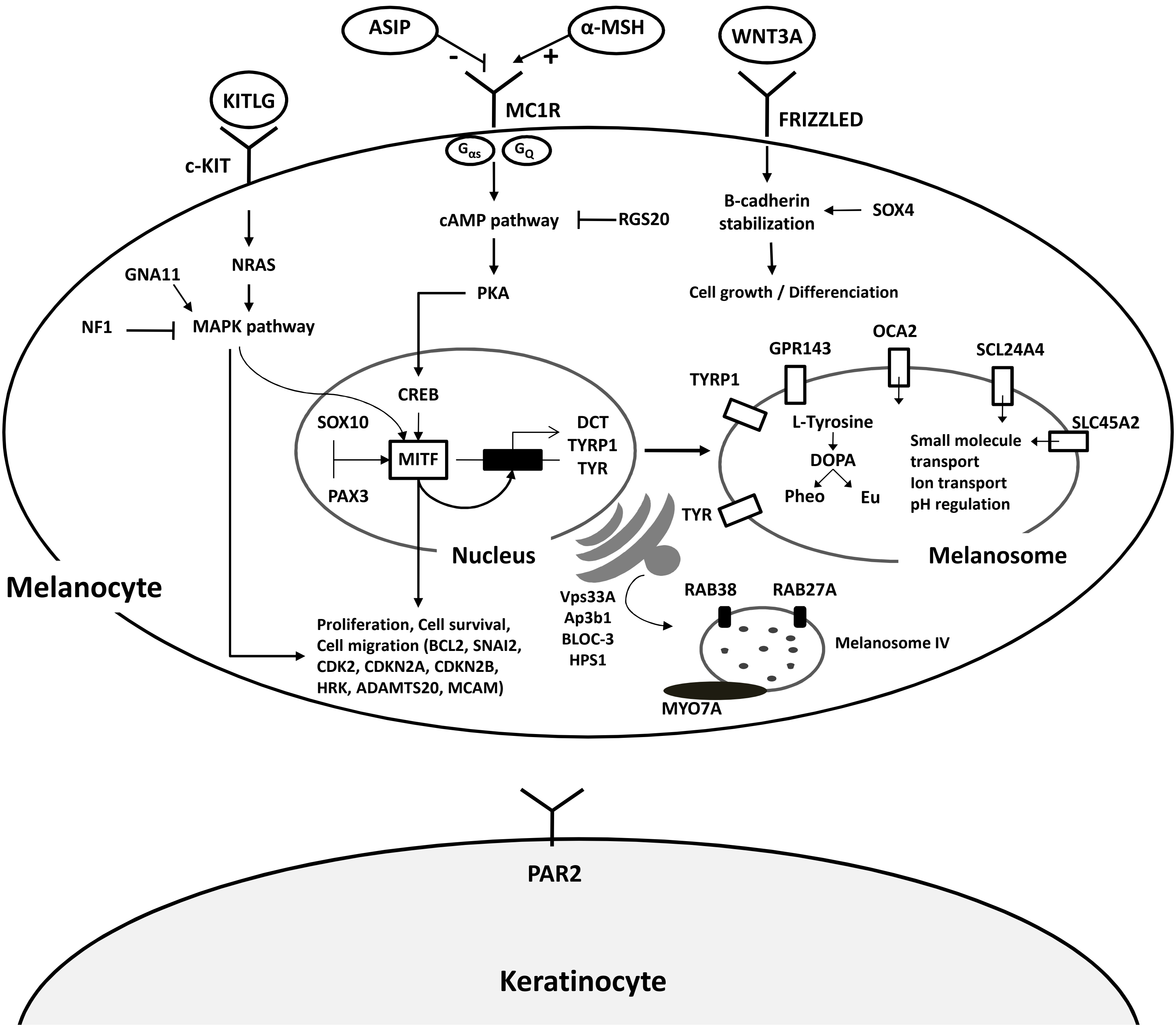

Figure S2. A selection of genetic factors affecting pigmentation and sun sensitivity in humans. Graph showing a selection of genes involved in melanocyte development, melanin synthesis, and melanosome biogenesis, transport and transfer.
